# Oral Health in Deaf and Hard-of-Hearing Individuals: Addressing Widespread Global Health Barriers Through Emerging Digital Technologies—A Comprehensive Review

**DOI:** 10.3390/dj14090589

**Published:** 2026-09-11

**Authors:** Maria Pia Di Palo, Federica Di Spirito, Francesco Giordano, Giuseppina De Benedetto, Elena Gaia Colacurcio, Vittoria Ferraro, Olimpia Prevete, Antonio Di Giulio, Marina Garofano, Alessia Nunziante, Alessia Bramanti

**Affiliations:** 1 Department of Medicine, Surgery and Dentistry, University of Salerno, 84081 Baronissi, Italy; frgiordano@unisa.it (F.G.); giusydb15@gmail.com (G.D.B.); e.colacurcio@studenti.unisa.it (E.G.C.); v.ferraro6@studenti.unisa.it (V.F.); o.prevete@studenti.unisa.it (O.P.); a.digiulio1@studenti.unisa.it (A.D.G.); mgarofano@unisa.it (M.G.); alenunziante@unisa.it (A.N.); abramanti@unisa.it (A.B.); 2 Department of Innovative Technologies in Medicine & Dentistry, University “G. d’Annunzio” Chieti-Pescara, 66100 Chieti, Italy

**Keywords:** oral health, oral diseases, oral status, deaf, hearing loss, hard-of-hearing, emerging technology, artificial intelligence, virtual reality, augmented reality

## Abstract

**Background/Objectives:** Deaf or Hard-of-Hearing (DHH) individuals face significant barriers in accessing healthcare, which may negatively impact oral health outcomes. Despite increasing recognition of these disparities, oral health remains underexplored, and the integration of emerging digital technologies to support accessible dental care is still limited. Therefore, the present comprehensive review aims to provide an overview of the oral health status of DHH individuals and the potential role of emerging digital technologies specifically tailored for the DHH population, considering, when relevant, the heterogeneity of this population based on the subgroup impairments classification. **Methods:** An electronic search was conducted across PubMed/MEDLINE, Scopus, and Web of Science databases till October 2025. An additional manual search was performed through reference screening of included studies. **Results:** DHH individuals exhibited poor periodontal health, high caries experience with predominant untreated decayed components, and unmet orthodontic needs. Emerging technologies, including m-health applications, 3D sign language avatars, speech-to-text systems, and virtual reality, may improve oral health literacy, reduce dental anxiety, and enhance patient-provider communication. **Conclusions:** Disparities in oral health among DHH individuals stem primarily from communicative impairments. Integrating tailored digital solutions into clinical pathways and professional education may offer a viable solution for fostering equitable, patient-centered dental care.

## 1. Introduction

The World Health Organization (WHO) defines disability as a global public health and human concern [1], which constitutes a priority condition for societal development [2]. Indeed, disability relates to a degree of functional impairment that interferes with major life activities [3].

Individuals with a disability represent a highly vulnerable group, considering they face barriers that can impair systemic [4] and oral health [5], constituting a special healthcare need (SHCN) populations widely heterogeneous, with differences in health maintenance capabilities based on the severity of impairments and cognitive levels [6]. As a consequence of this heterogeneity, several classifications have been proposed, including those of Frank and Winter, which identified eight impairment categories: cognitive, behavioral (e.g., maladjustments), sensory (e.g., blindness, deafness), physical, and speech-related [6].

Within the broad spectrum of disabilities recognized by the WHO, hearing loss was defined as the inability to hear sounds at 20 decibels or more in both ears and encompasses a wide spectrum of severity, ranging from mild impairment to profound deafness [1].

The WHO 2025 estimates that hearing loss is a global health concern, affecting about 430 million subjects worldwide, including 34 million children, and accounting for more than 5% of the global population requiring rehabilitation interventions [1].

Beyond the sensory impairments, hearing loss may affect various aspects of life. It poses limitations in communication and speech, contributes to social isolation, loneliness, and stigma, negatively impacts cognition, and significantly affects both society and the economy [1]. Moreover, it influences years lived with disability and disability-adjusted life years, and severely restricts access to education and employment [1].

Subjects with hearing impairment, particularly those who are Deaf or Hard-of-Hearing (DHH), often encounter substantial barriers in accessing healthcare services, among which communication difficulties between patients and healthcare providers represent one of the most critical challenges [2,3]. Taken together, these barriers result in reduced healthcare access—including in the dentistry setting—limited understanding of medical information, and consequently poorer health outcomes [2,3].

Oral health represents a fundamental component of well-being, systemic health, and quality of life [3,7]. However, it remains one of the most underestimated areas within healthcare [8], particularly in the pediatric population [7], and even more among individuals affected by disabilities [3,9].

Moreover, oral health appeared to be disproportionately affected in populations with disabilities, including those with hearing impairment [4,5,10,11,12]. In fact, evidence highlighted that DHH individuals tend to exhibit poorer oral health status, higher prevalence of dental diseases, and reduced access to preventive and therapeutic dental care [3].

Contributing factors included communication barriers, limited health literacy regarding specific needs, both among DHH individuals and healthcare providers, socioeconomic constraints, and inadequate adaptation of healthcare services [2,3,9].

Importantly, the heterogeneity of hearing loss in terms of severity, etiology, age of onset, and rehabilitative pathways may further influence patients’ ability to access and benefit from oral healthcare services [13,14]. Therefore, a comprehensive understanding of the clinical and functional characteristics of hearing loss is essential to contextualize the challenges encountered in dental settings and to develop effective, patient-centered strategies of care.

In this context, recent advances in digital assistive technologies have emerged as promising tools for overcoming the barriers that hinder optimal access to and quality of care for the SHCN population [15,16,17,18,19]. These include visual-based educational platforms, mobile health applications, teledentistry approaches, real-time speech-to-text systems, and sign language–supported communication tools, which may enhance patient–provider interaction, promote oral health literacy, and facilitate adherence to preventive and therapeutic interventions [15,16,17,18,19].

However, despite the growing recognition of health disparities affecting individuals with disabilities, oral health in DHH individuals remains insufficiently explored and often underestimated in clinical practice and research. Furthermore, although technological solutions are becoming increasingly widespread, they often remain isolated and poorly integrated into dental care pathways, particularly for individuals with SHCN who have specific requirements—such as DHH individuals—which differ from the needs of other SHCN [11,20,21].

In this regard, the lack of tailored implementation and the limited alignment between technological tools and the communicative and clinical needs of DHH patients may further contribute to persistent inequalities in access and quality of dental care [3,4,10,11,18].

Considering the WHO projections, which estimated that by the year 2050, it is expected that almost 2.5 billion people will have some degree of hearing loss, and over 700 million people will have hearing loss [1], addressing these disparities, the underlying causes, and the potential role of currently available emerging digital tools in overcoming these barriers represents a public health need.

Therefore, the present comprehensive review aims to provide a comprehensive overview of the oral health status of DHH individuals and the current and potential role of emerging digital technologies specifically tailored for DHH individuals. By integrating evidence on clinical oral status, barriers, and technological innovations, this review aimed to discuss a structured framework to support more inclusive, accessible, and patient-centered dental care strategies in the digital era, considering, when relevant, the heterogeneity of this population based on the subgroup impairments classification (e.g., degree of hearing loss, age of onset).

## 2. Materials and Methods

### 2.1. Search Strategy

To perform the present comprehensive review, an electronic search was conducted on 21 October 2025 in the following databases: PubMed/MEDLINE, Scopus, and Web of Science.

No lower date limit was applied, and the search included all records indexed in the databases from their inception up to 21 October 2025.

The same search strategy was conducted across the databases mentioned above, using a combination of keywords related to hearing conditions, oral health status, and digital technologies. Full database-specific search strings and syntax are provided in Appendix A.

No restrictions or filters were used in any databases to conduct the electronic search.

An additional manual search was performed through the screening of the bibliography of the studies considered included from the electronic search, to minimize the possibility that relevant studies may have been overlooked.

Version 2.142.0 of the Mendeley Reference Manager tool was utilized to manage references.

### 2.2. Study Selection and Eligibility Criteria

**Figure 1:**
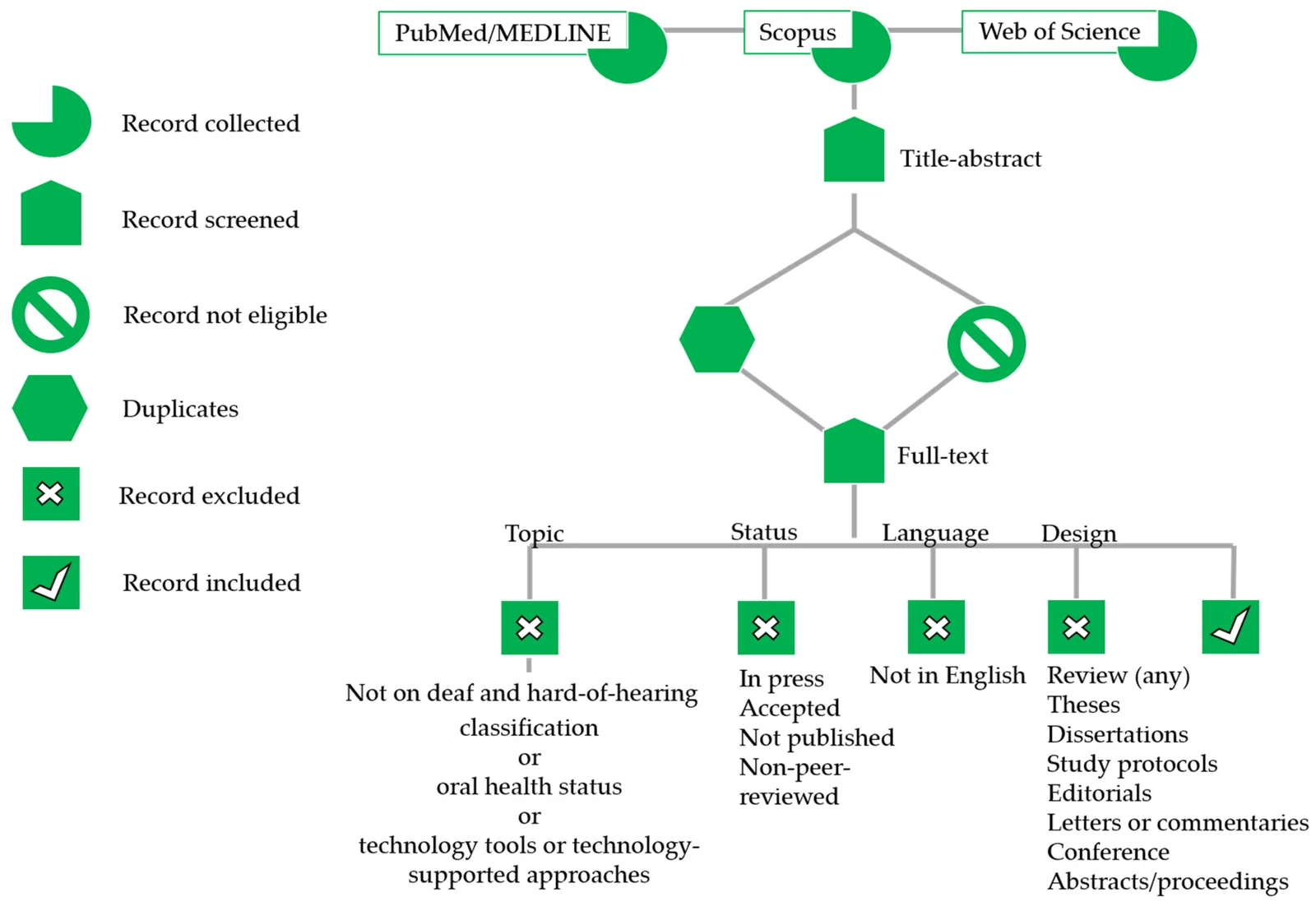
Schematic representation of the study selection process, illustrating the main steps from the identification of records through the electronic search to the final inclusion of eligible studies, including the removal of duplicates and the exclusion of records not meeting the predefined eligibility criteria.

The records retrieved from the electronic search were collected and screened by two independent reviewers (M.P.D.P. and G.D.B.), and in case of disagreement, a third reviewer was involved (F.D.S.). Duplicates in different databases were considered once, while records with title-abstracts not relevant were not read in full text, as done for the remaining records. Of these, records were excluded if not compliant with the eligibility criteria and were included otherwise. A schematic illustration of the study selection process is displayed in Figure 1.

The inclusion criteria were: case reports, case series, case–control studies, cross-sectional studies, randomized clinical trials, retrospective/prospective studies conducted in human populations and published in English. Eligible studies had to concern DHH individuals and address at least one aspect of the following review objectives: (i) DHH classification; (ii) oral health status; (iii) available emerging technology tools or technology-supported approaches for this population. Depending on the review objective, the relevant exposure, intervention, or topic of interest was considered, with no restrictions on the clinical or healthcare setting. Studies assessing mixed-disability cohorts were considered eligible only if data for DHH individuals were reported separately.

The exclusion criteria were: narrative, scoping, and systematic reviews; conference abstracts or proceedings without a full peer-reviewed article, theses and dissertations, study protocols, editorials, letters, commentaries, and other non-peer-reviewed publications; studies not published, in press, or accepted; published studies not in English; and articles not focused on at least one of the aforementioned main topics or for which data specific to DHH individuals could not be identified separately.

No age restrictions were applied as part of the eligibility criteria; therefore, studies involving DHH individuals of any age were considered eligible.

### 2.3. Data Extraction, Collection, and Synthesis

Data from the relevant studies included from the electronic and manual search were extracted and collected using Microsoft Excel software 2019 (Microsoft Corporation, Redmond, WA, USA).

A narrative qualitative synthesis was separately performed to identify the current DHH classifications, oral health status, and the available emerging technology tools.

Regarding the oral health status, the following domains were analyzed, and the following data were extracted:

- Periodontal health and disease parameters: plaque index, gingival index, probing depth, bleeding on probing, clinical attachment level, simplified oral health index, and community periodontal index;
- Dental caries parameters: decayed, missing/extracted, filled, teeth/surface domains;
- Oral manifestations and dental anomalies;
- Malocclusions and orthodontic status: dental and skeletal class; transverse dental relationship, overjet, overbite, impacted teeth, spacing/diastema, crowding, rotation, and the dental aesthetic index.

To provide a descriptive overview of the clinical data, summary prevalence values were calculated as descriptive proportions, defined as the total number of affected individuals divided by the total number of individuals evaluated across studies reporting the corresponding outcome. For continuous clinical indices (such as PI, GI, OHI-S, CAL, and DMFT/dmft), descriptive summary values were calculated as sample-size-weighted means of study-level means, considering studies reporting comparable metrics. These calculations were intended solely to provide a descriptive synthesis and were not considered meta-analytic estimates. To account for clinical and methodological heterogeneity across the included literature, these summary values are presented alongside study-specific characteristics, age ranges, and individual findings detailed in the extraction tables.

Only data that adhered to the established eligibility criteria were included in the extraction process; for studies involving mixed-disability cohorts, only data specifically about DHH individuals were extracted, while data concerning other disability subgroups were excluded.

## 3. Results

### 3.1. Definition and Classification of Hearing Loss

Hearing loss was defined as the inability to hear sounds at 20 decibels or more in both ears, and, in particular, disabling hearing loss as a hearing disorder greater than 35 decibels in the better hearing ear [1].

The main classification criteria are summarized below, while additional details are available in Supplementary File S1.

#### 3.1.1. Classification Based on Severity

Hearing loss can be classified based on severity into five categories: mild, moderate, moderately severe, severe, and profound [1]. Individuals with mild-to-moderate hearing loss were typically referred to as “hard-of-hearing”, while those with severe or profound hearing loss were more commonly described as “deaf” [1,22].

#### 3.1.2. Classification Based on Anatomic Defect

In relation to anatomic defects, hearing loss can be divided into three categories: conductive hearing loss (CHL), sensorineural hearing loss (SNHL), and mixed hearing loss [23]. The audiological characteristics are provided in Supplementary File S1.

#### 3.1.3. Classification Based on Age of Onset and Time of Onset in Relation to Language Development

Numerous factors may contribute to the onset of hearing loss, which can occur at different stages throughout the lifespan (during the prenatal period, perinatal, childhood and adolescence, adulthood, and older age) and can be distinguished into: congenital, prelingual, and postlingual hearing loss, which have significant implications for language development and communication outcomes [1].

The causes associated with hearing loss throughout the different stages of lifespan are detailed in Supplementary File S1.

#### 3.1.4. Classification Based on Etiology

Based on etiology, hearing loss can be distinguished into two major categories: genetic or nongenetic (acquired) [23].

Hearing loss of genetic etiology can be further categorized into simple Mendelian inheritance and complex inheritance [23].

Mendelian forms are further classified as syndromic or nonsyndromic, depending on whether additional anomalies are present [23]. Both syndromic and nonsyndromic types are then subdivided according to their mode of inheritance: autosomal dominant, autosomal recessive, X-linked, or mitochondrial [23,24,25].

Comprehensive details regarding hearing loss in relation to inheritance patterns and both syndromes an non-syndromic forms of hearing loss are presented in Supplementary File S1.

Indeed, around 36% of individuals over 75 years present with some degree of hearing impairment; in fact, the incidence of hearing loss increases significantly with age [25]. Among these cases, nearly one-third are considered acquired, another third are attributed to genetic inheritance, while the remaining third has an undetermined etiology [25].

Different factors and conditions were associated with nongenetic (acquired) hearing loss, such as drugs [23,26], infection [23,27,28], noise [29,30,31], presbycusis [14,23,25,32], and otosclerosis [23,33,34,35,36].

Specific characteristics of acquired hearing loss associated with different factors or conditions are detailed in Supplementary File S1.

#### 3.1.5. Other Classification

Hearing loss may also be described according to its clinical course in stable, progressive, fluctuating, or sudden, the range of frequencies affected (low, high, or across all frequencies), and the laterality of involvement (unilateral or bilateral) [1].

Together, these classification systems provide a comprehensive framework for understanding the heterogeneity of hearing impairment and its clinical implications.

**Figure 2:**
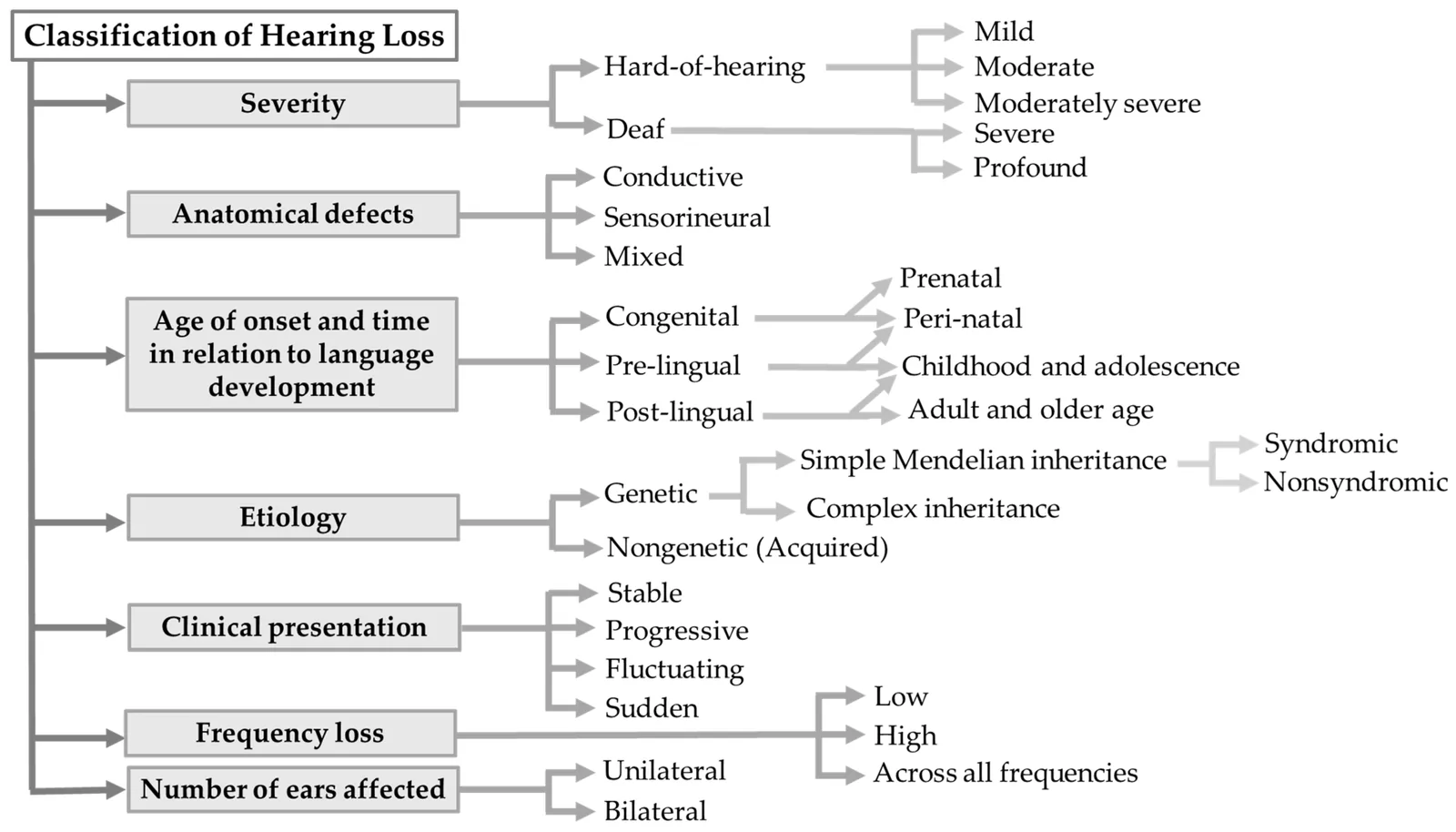
Classification of hearing loss based on severity, anatomical defects, age of onset and time in relation to language development, etiology, clinical presentation, frequency loss, and number of ears affected.

Figure 2 summarizes the classification of hearing loss.

### 3.2. Oral Health Status Among DHH Individuals

Current evidence suggests that individuals with disabilities tend to experience health disparities [3]. Indeed, individuals with disabilities encounter several barriers to health care accessibility, consequently resulting in suboptimal healthcare outcomes [2,3,9].

Specifically, DHH individuals encounter difficulties both in independently maintaining their oral health and in effectively receiving appropriate professional treatment. In fact, several studies have highlighted that DHH individuals tend to exhibit poorer oral health status, thus representing a population whose dental needs are largely unaddressed [3,5,7,8,9,37,38,39].

#### 3.2.1. Periodontal Health and Disease in DHH Individuals

Numerous studies have investigated the periodontal condition of the DHH individuals, noting a generally poor periodontal status with both a higher prevalence of gingivitis and periodontitis, as well as greater severity of periodontal disease.

Nineteen studies [5,37,38,40,41,42,43,44,45,46,47,48,49,50,51,52,53,54,55], which investigated periodontal health and disease among DHH individuals, were included, encompassing a total of 2804 subjects (age range 5–30 years).

Goud et al. [44] reported a statistically significantly higher frequency of healthy periodontium in DHH individuals compared with visually impaired children, and also Jain et al. [45] reported a worse Community Periodontal Index (CPI) than that of blind individuals. In relation to gender, Jnaneswar et al. [46], Rawlani et al. [50], and Shivakumar et al. [38] did not find statistically significant differences regarding CPI scores between males and females. Based on age, Kumar et al. [48] highlighted that CPI 1 (bleeding) was higher in the 12–17 subgroup compared with the 18–23 subgroup, while CPI 2 and CPI 3 were lower.

No statistically significant differences were found regarding CPI scores between males and females.

The prevalence of gingivitis was assessed in 380 DHH individuals, and was diagnosed in 205 subjects (53.94%). Avasthi et al. [41] showed that the prevalence of gingivitis was lower compared with subjects blind.

The prevalence of periodontitis was assessed in 149 DHH individuals, and was diagnosed in 34 subjects (22.8%). Tefera et al. [55] showed that individuals above the age of 18 years had a higher prevalence of periodontal disease (29.2%).

Plaque index (PI) was assessed in 716 DHH individuals, and the average was 1.13. Al-Maweri et al. [37] showed that the mean PI was lower compared with subjects blind (these findings were in contrast to those reported by Rezaei et al. [51]), with compound disabilities, cognitive impairments, or physical impairments. In relation to genders, Ghannam et al. [43] found that PI indices were higher among males compared to females, while Rawlani et al. [50] and Shivakumar et al. [38] showed no statistically significant difference. A significant positive correlation was found between the Pediatric Oral Health-Related Quality of Life and PI [43].

Gingival index (GI) was assessed in 489 DHH individuals, and the mean was 1.15. Al-Maweri et al. [37] showed that the mean GI was lower compared with subjects blind (these findings were in contrast to those reported by Rezaei et al. [51]), with compound disabilities, cognitive impairments, or physical impairments. In relation to genders, Ghannam et al. [43] found that the GI index was higher among females compared to males, while Rawlani et al. [50] showed no statistical differences. A significant positive correlation was found between the Pediatric Oral Health-Related Quality of Life and GI [43]. Based on age, Samal et al. [53] reported that no statistically significant difference in gingival bleeding was found in relation to age.

Clinical attachment level (CAL) was assessed in 287 DHH individuals, and the average was 0.29. Rawlani et al. [50] found that the CAL index was statistically significantly higher among females compared to males, while Shivakumar et al. [38] found an opposite trend.

Simplified oral health index (OHI-S) was assessed in 923 DHH individuals, and the mean was 1.97. Gaçe et al. [42] showed that the mean OHI-S score was statistically significantly different when compared among the different disability groups: higher than that of individuals with Down syndrome, cognitive impairment, blindness (also reported by Goud et al. [44] and Reddy et al. [52]), autism, and cerebral palsy. Instead, Kumar et al. [48] reported that no statistically significant difference was found between age subgroups for the OHI-S index. In relation to gender, Rawlani et al. [50] found that the OHI-S index was statistically significantly higher among females compared to males, while Shivakumar et al. [38] found an opposite trend.

Table 1 summarizes the periodontal status among DHH individuals.

#### 3.2.2. Dental Caries Among DHH Individuals

Although dental care and treatment have undergone significant advancement, dental caries remain a widespread public health challenge worldwide, affecting both children and adults [3,4]. Several studies have highlighted that individuals with disabilities have a greater risk and higher prevalence of dental caries [2,3,4,7,9,10,20,37,38,39,54,56,57].

The prevalence of caries was assessed in 1366 DHH individuals, and was diagnosed in 833 subjects (60.98%). In comparison with other types of impairments, Avasthi et al. [41] found that the prevalence of dental caries in DHH individuals was higher compared with that of the blind group, while Vyavahare et al. [58] and Wei et al. [59] found that it was higher than that of healthy children.

Based on age, Jain et al. [45] found an increased prevalence with age, while Samal et al. [53] recorded that dental caries prevalence was not significantly associated with age. Specifically, Sandeep et al. [5] highlighted that a statistically significant difference was reported among age groups between the 9–12 years old vs. the 13–16 years old group, and between the 6–8 years old group vs. the 13–16 years old group. No statistically significant difference was reported between groups 6–8 vs. 13–16 years old.

In relation to gender, Suma et al. [9], Wei et al. [59] and Samal et al. [53] not reported a statistically significant difference in the gender distribution of the caries disease.

Instead, Samal et al. [53] highlighted that a worsened level of hearing loss was associated with a significantly higher prevalence of dental caries, while Tefera et al. [55] found a significant association with grade level, caregivers’ support, or tooth brushing, oral health status, or medication intake.

The Decayed, Missing, and Filled Teeth (DMFT) index was assessed in 4039 DHH individuals, and the mean was 1.97. Several studies identified the Decayed domain as the main statistically significant contributor to the total DMFT index [38,45,60]. In contrast, the Missing and Filled domains generally did not account for a major proportion of the DMFT. Only Shaw et al. [54] identified the Filled component as the primary contributor to the DMFT index, and Wei et al. [59] reported that only the Missing and Filled components were significantly higher in DHH individuals compared to healthy controls. Age-related differences were also observed: Jnaneswar et al. [46] reported a statistically significant increase in the Missing domain in the 11–15 years subgroup compared to the 5–10 years subgroup; while Jain et al. [45] highlighted that across age subgroups, Missing and Filled domains were higher only in the group aged 18–22 years, where their contribution was more pronounced.

Across the included studies, the mean DMFT values observed among DHH individuals showed a heterogeneous pattern when compared with other disability groups. In relation to visually impaired or blind individuals, results were inconsistent: several studies reported slightly higher DMFT scores in DHH individuals subjects [44,52,60,61], whereas others found lower or comparable values [37,42,62], with some differences not reaching statistical significance.

When compared with individuals with cognitive or intellectual impairments, a more consistent trend emerged, as most studies (including Chand et al. [61], Gaçe et al. [42], and Hashemi et al. [39]) reported lower DMFT values in DHH individuals. Similarly, comparisons with individuals with cerebral palsy generally indicated lower DMFT scores in DHH individuals [42,61,63].

In contrast, findings were less uniform when considering individuals with physical or orthopedic impairments. Some studies suggested slightly higher DMFT values among DHH individuals [44], whereas others reported lower values [64], or no statistically significant differences [39].

Comparisons with individuals with multiple or compound disabilities also yielded mixed results, with both higher [37] and lower [64] DMFT scores in DHH individuals.

Regarding other specific conditions, such as autism and epilepsy, the available evidence suggested that DHH individuals may exhibit higher DMFT values than those with autism [42,54], but lower values compared with epilepsy [54]. Additionally, DMFT scores in DHH individuals were generally lower than those reported for individuals with Down syndrome [39,60], which consistently showed some of the highest values across disability categories.

Finally, when compared with healthy individuals, most studies [58,59] indicated higher DMFT scores among DHH individuals, although these differences were not always statistically significant.

In relation to gender, Ghannam et al. [43], Rawlani et al. [50], and Vyavahare et al. [58] did not show a statistically significant difference in DMFT.

Vyavahare et al. [58] highlighted a statistically significant association between DMFT score and socioeconomic status, which was not found by Jain et al. [45], even with caste, level of education, or degree of hearing loss.

Based on age, Jain et al. [45], Aruna et al. [65], Sandeep et al. [5], and Sanjay et al. [62] showed that there was a significant increase in the DMFT for the advancing age subgroup.

A significant positive correlation was found between the Pediatric Oral Health-Related Quality of Life and DMFT [43].

The decayed, missing, and filled teeth (dmft) index was assessed in 2101 DHH individuals, and the mean was 1.61. Sandeep et al. [5] identified that the Decayed (also reported by Shyama et al. [60]) and the Missing domains were the main statistically significant contributors to the total DMFT index.

The mean dmft score in DHH individuals shows heterogeneous trends when compared with other disability groups. Several studies reported higher dmft compared with the visually impaired or blind, with statistically significant differences [52,62], and also compared with individuals with multiple and orthopedic impairments [64].

In contrast, other findings indicate comparable or lower dmft scores: Jain et al. [66] reported no statistically significant differences compared with other disability groups, including blind individuals, while Al-Maweri et al. [37] observed lower dmft values compared with physically impaired individuals but higher values than in subjects with compound disabilities, cognitive impairments, or blindness. Similarly, Nqcobo et al. [63] found lower dmft values compared with cerebral palsy, but higher values than in individuals with learning and mental disabilities.

In relation to gender, Ghannam et al. [43] found that the dmft scores were similar between the female and male subgroups.

Based on age, Jain et al. [45] highlighted that the dmft in the 5–7 age subgroup had significantly the highest values.

A significant positive correlation was found between the Pediatric Oral Health-Related Quality of Life and dmft [43].

The Decayed, Extracted due to caries, Filled Teeth (DEFT) was assessed in 540 DHH individuals, and the mean was 0.15. Age-related differences were observed: Jnaneswar et al. [46] identified the Decayed and Extracted domains as statistically significant contributors to the total DEFT index, with higher values reported in the 5–10 years subgroup compared to the 11–15 years subgroup.

The Decayed, Missing, and Filled Surface (deft) was reported in 302 DHH individuals, and the mean was 1.79. The mean deft score observed among DHH individuals showed a heterogeneous trend when compared with other disability groups. In particular, Chand et al. [61] reported that the DHH individuals exhibited the highest mean deft score compared to individuals with cognitive impairment, visual impairment, and cerebral palsy. In contrast, Gaçe et al. [42] reported that in deaf-mute individuals, lower deft values were found than those with cerebral palsy or blindness, but higher values compared to individuals with Down syndrome.

The Decayed, Missing, and Filled Surfaces (DMFS) index was assessed in 722 DHH individuals, and the mean was 6.22. In comparative analyses, Al-Maweri et al. [37] reported that the mean DMFS was lower than that observed in individuals with compound disabilities and cognitive impairment, but higher than that of blind and physically impaired subjects. Similarly, Shyama et al. [60] found that DHH individuals exhibited significantly higher DMFS values than visually impaired subjects, ranking second only to individuals with Down syndrome among the disability groups considered.

Shivakumar et al. [38] highlighted that lesion severity, as assessed by DMFS, often involved multiple affected surfaces per tooth. Trimeridou et al. [67] identified a positive correlation between the severity of hearing impairment and DMFS, although subgroup analysis showed that older adolescents with profound hearing loss had lower DMFS values compared with younger children presenting severe or moderate hearing loss.

With regard to demographic and behavioral variables, no statistically significant associations were found between DMFS and age or gender, nor between individual DMFS components (Decayed, Missing, or Filled Surfaces) and these factors. Furthermore, no significant relationship emerged between DMFS and dental fear or anxiety.

The Decayed, Missing, and Filled Surface (dmfs) was assessed in 572 DHH individuals, with a mean of 9.54. Age-related differences were also observed: specifically, Jain et al. [45] reported that the 5–7 years subgroup showed significantly higher dmfs values compared to older age groups, whereas Trimeridou et al. [67] provided specific data for the 11-year subgroup. The dmfs observed among DHH individuals showed a heterogeneous trend when compared with other disability groups. In particular, Al-Maweri et al. [37] reported that dmfs in DHH individuals were lower than those observed in subjects with physical and cognitive impairments, but higher compared to individuals with compound disabilities and blindness.

Table 2 summarizes the dental caries status among DHH individuals.

#### 3.2.3. Oral Manifestations and Dental Anomalies Among DHH Individuals

Individuals with special healthcare needs, including those with hearing impairments, may experience an increased lifetime risk of oral diseases and manifestations, with conditions affecting the dental hard tissues representing the most frequently reported findings [11,43]. DHH individuals may exhibit dental anomalies, especially within syndromic and genetically determined forms of hearing impairment, including alterations in tooth number, morphology, and structure, potentially contributing to a more complex oral health profile in affected individuals [44,45].

However, evidence regarding oral mucosal manifestations in DHH individuals remains extremely limited. To date, no studies have addressed the prevalence or clinical characteristics of oral mucosal pathologies in this population, highlighting a relevant gap in the current literature.

Four studies [7,11,24,46], which investigated dental anomalies among DHH individuals, were included, encompassing a total of 481 subjects (age range 5–24 years).

Dental structural anomalies, such as dental fluorosis, were examined in 268 DHH individuals and were found in 50 subjects (18.66%).

Dental anomalies in shape and volume were examined in 277 DHH individuals and were found in 43 teeth (15.52%). A statistically significant correlation was found between dental shape anomalies and fluorosis [8].

Dental anomalies involving tooth number were examined in 274 DHH individuals, and 27 cases of dental agenesis (9.85%) were found, while no supernumerary teeth were found [7,46]. Only one subject had more than three congenitally missing teeth [8], and the upper lateral incisor agenesis was the most prevalent recorded.

Table 3 summarizes the dental anomalies retrieved among DHH individuals.

#### 3.2.4. Malocclusions and Orthodontic Needs Among DHH Individuals

Special healthcare needs individuals, including those with hearing impairments, tend to exhibit higher rates of dental problems, such as malocclusion and orthodontic needs. Consistent with the previously aforementioned issue of suboptimal dental care in individuals with disabilities, the need for orthodontic treatment is also frequently overlooked [8]. In particular, several studies have highlighted that individuals with disabilities have a greater risk and higher prevalence of malocclusions and orthodontic needs.

Previous studies have specifically evaluated the prevalence of malocclusions and orthodontic needs in DHH individuals.

Six studies [8,40,41,68,69,70], which investigated the orthodontic bite status among DHH individuals, were included, encompassing a total of 755 subjects (age range 5–30 years). Premeswari et al. [69] found that the majority of subjects presented with moderate-to-severe malocclusions, suggesting a high need for orthodontic treatment, while Sokolohorska-Nykina et al. [8] reported no significant correlation between the severity of hearing loss and the type of malocclusion. Finally, Avasthi et al. [41] compared a higher prevalence of malocclusions in DHH individuals (57.98%) with that of the blind group (30.69%).

Skeletal classes were examined in 33 DHH individuals, and no significant skeletal abnormalities were observed: class I was found in 27 (82.00%), class II in 3 (9.00%), and class III in 3 (9.00%).

Dental classes were examined in 390 DHH individuals, recording class I for 285 subjects (73.08%), class II for 83 (21.28%), and class III for 22 (5.64%). The class II division was specified 47 of the 83 cases as follows: 34 cases had class II division I, and 13 class II division II. A significant correlation was found between class II dental malocclusions and concomitant systemic conditions by Sokolohorska-Nykina et al. [8].

Transverse relationships were examined in 281 DHH individuals and were normal in 216 (76.87%), crossbite in 8 (2.85%), and brodiebite in 6 (2.13%).

Overjet (≥3 mm) was assessed in 68 DHH individuals and was found in 27 (39.7%). The prevalence of increased overjet was statistically lower in DHH individuals when compared with subjects with cognitive impairments [70].

Overbite was assessed in 342 DHH individuals and was reported as normal in 184 (53.80%), deep bite 108 (31.58%), and openbite in 50 (14.62%). In particular, openbite was found in the anterior region in 35 (70%) of cases, posterior in 9 (18%), and both anterior and posterior in 6 (12%). Statistically significant differences between genders were found in the overbite distribution by Ciger et al. [68].

Impacted teeth were examined in 213 DHH individuals and were found in 15 (7.04%) for a total of 22 impacted teeth (20 upper canines, 1 lower canine, and 1 lower second premolar).

Spacing/diastema was examined in 390 DHH individuals and was found in 59 (15.13%).

Crowding was examined in 274 DHH individuals and was found in 88 (32.12%), in particular, slight in 19 (6.93%), moderate in 8 (2.92%), severe in 17 (6.20%), and not specified in 44 (16.06%) [8,68]. Dental crowding was significantly associated with systemic health status by Sokolohorska-Nykina et al. [8].

Rotation was assessed in 116 DHH individuals and was found in 10 (8.6%).

The Dental Aesthetic Index (DAI) was assessed in 129 DHH individuals, of whom 69 (53.49%) children reported a need for treatment with a DAI score ≥ 26; 18 subjects (26.23%) fell into the “mandatory treatment” category (DAI ≥ 36). Although Sokolohorska-Nykina et al. [8] registered that the severity of malocclusion tends to increase slightly with age (rising from a DAI of 31.2 to 34.88), but this difference was not statistically significant.

Table 4 summarizes the orthodontic bite status among DHH individuals.

**Table 3:** Dental anomalies in DHH individuals. Abbreviations: Deaf and Hard-of-Hearing (DHH).

Study	Country	DHH Population Characteristics	Dental Anomalies	Main Results and Statistically Significant Findings
Alyami et al., 2022 Cureus [40]	Saudi Arabia	Sample size: 116 Age: 5–16 years Target population: DHH children in Jeddah city; no control	Mild fluorosis: 32	In about 27.58% of deaf and hard-of-hearing children, mild fluorosis was recorded.
Ciger et al., 2010 Eur J Dent [68]	Turkey	Sample size: 213 Age: 16.37 ± 2.53 years, age range: 10–24 years Target population: DHH students at the special needs schools of Ankara; no control	Malformed teeth: 16 upper lateral incisors; 4 upper central incisors; 4 upper second molar; 4 lower central incisors; 2 lower lateral incisors; 2 upper canines; 2 upper first premolar; 1 lower first premolar; 2 lower second molar Dental agenesis: 11 upper lateral incisors; 6 lower second premolars; 2 upper central incisors; 2 lower central incisors; 1 upper second premolar	In total, 37 cases of malformed teeth were retrieved in 16 individuals (7.5%). One male had 8 malformed teeth. In total, 22 cases of dental agenesis were retrieved in 13 individuals (6.0%), but none had more than three congenitally missing teeth. No supernumerary teeth were found.
Samal et al., 2024 Cureus [53]	India	Sample size: 91 Age: 15.45 ± 2.51 Target population: DHH students of 10 schools; no control	Fluorosis: 7 (7.7%)	In about 7.7% of DHH children, fluorosis was recorded.
Sokolohorska-Nykina et al., 2025 Stomatological Bulletin [8]	Ukraine	Sample size: 61 Age: range 7–20 years Target population: DHH students at the special school for DHH in Poltava; no control	Fluorosis: 11 (18.03%) Dental shape anomalies: 3 (4.92%) Dental size anomalies: 3 (4.92%) Dental agenesis: 5 (8.2%)	A statistically significant correlation was found between dental shape anomalies and fluorosis. Upper lateral incisor agenesis was the most prevalent. No cases of canines, first premolars, or first or second molar agenesis were found. One subject had more than three congenitally missing teeth. No supernumerary teeth were found.

**Table 4:** Malocclusions and orthodontic status in DHH individuals. Abbreviations: Deaf and Hard-of-Hearing (DHH), Dental Aesthetic Index (DAI); not defined (N/d); millimeters (mm).

Study	Country	DHH Population Characteristics	Malocclusions and Orthodontic Status	Main Results and Statistically Significant Findings
Alyami et al., 2022 Cureus [40]	Saudi Arabia	Sample size: 116 Age: 5–16 years Target population: DHH children in Jeddah city; no control	Class I: 80 (68.97%) Class II division I: 20 (17.24%) Class II division II: 11 (9.48%) Class III: 5 (4.31%) Spacing: 17 (14.65%) Rotation: 10 (8.6%)	In about 31.03% of DHH children, a malocclusion status of class II–III was recorded.
Avasthi et al., 2011 Int J Dent Clin [41]	India	Sample size: 264 Age: range 5–16 years Target population: DHH students at special needs schools in Delhi and Gurgaon; control with other special needs students at the same schools	Malocclusions were detected in 57.98% of subjects.	The prevalence of malocclusions in DHH individuals was higher compared with that of the blind group (30.69%).
Ciger et al., 2010 Eur J Dent [68]	Turkey	Sample size: 213 Age: 16.37 ± 2.53 years, age range: 10–24 years Target population: DHH students at the special needs schools of Ankara; no control	Class I: 160 (75.00%) Class II: 36 (17.00%) Class III: 17 (8.00%) Normal overbite: 51 (23.9%) Deep Bite: 82 (38.4%) Open Bite: 50 (23.4%) Anterior openbite: 35 (16.4%) Posterior openbite: 9 (4.2%) Anterior and posterior: 6 (2.8%) Transverse relationship Normal: 148 (69.4%) Crossbite: 8 (2.3%) Brodiebite: 6 (1.9%) Crowding Slight: 19 (8.9%) Moderate: 8 (3.7%) Severe: 17 (18.3%) Diastema: 40 (18.7%) Impacted teeth: 20 upper and 1 lower canines; 1 lower second premolar	Most of the subjects had Class I malocclusion, while a high prevalence of deep bite and open bite was observed. Statistically significant differences between genders were found in the overbite distribution. In total, 22 cases of impacted teeth were retrieved in 15 individuals (7.04%).
Dinesh et al., 2003 Int Dental J [70]	India	Sample size: 68 Age: range 11–30 years Target population: DHH students of eight special needs schools in the South Canara district of Karnataka State; controls were other special needs students at the same schools	Overjet (≥3 mm): 27 (39.7%) Anterior openbite: 0 (0.00%) Anterior crossbite: 0 (0.00%)	The prevalence of increased overjet was statistically lower in DHH when compared with subjects with cognitive impairments. The reported need for treatment was 29.41% for children with definite malocclusion (DAI score 26–30), 2 (2.94%) with severe (DAI score 31–35), and 2 (2.94%) with very severe malocclusion (DAI score ≥ 36), while 44 (64.71%) had no malocclusions.
Prameswari et al., 2022 Eur J Dent [69]	Indonesia	Sample size: 33 Age: 8–12 years Target population: DHH students at the special needs schools in Surabaya; no control	Skeletal class Class I: 27 (82.00%) Class II: 3 (9.00%) Class III: 3 (9.00%) Malocclusion: 33 (100.00%) Malocclusion severity: Grade 2 (mild): 3 (9.00%) Grade 3 (moderate): 12 (36.00%) Grade 4 (severe): 12 (36.00%) Grade 5 (very severe): 6 (19.00%)	No significant skeletal abnormalities were observed, potentially indicating that DHH individuals may not appear to be affected by craniofacial growth. A dentoalveolar discrepancy was reported, with protrusion of the upper incisors and retrusion of the lower incisors. The majority of subjects presented with moderate-to-severe malocclusions, suggesting a high need for orthodontic treatment.
Sokolohorska-Nykina et al., 2025 Stomatological Bulletin [8]	Ukraine	Sample size: 61 Age: range 7–20 years Target population: DHH students at the special school for DHH in Poltava; no control	Class I: 45 (73.77%) Class II: 16 (26.23%) Class II division I: 14 Class II division II: 2 Class III: 0 (0.00%) Deep bite (>2 mm): 26 (42.62%) Crowding (in Class I): 44 (96.6%) Diastemas/Spacing: 2 (3.4%)	A significant correlation was found between sagittal malocclusions (Class II) and systemic conditions, while crowding was significantly associated with systemic health. No significant correlation was reported between the severity of hearing loss and the type of malocclusion, suggesting that hearing loss may not influence tooth position. The reported need for treatment was 73.76% of the children, with a DAI score ≥ 26; 16 subjects (26.23%) fell into the “mandatory treatment” category (DAI ≥ 36). Although the severity of malocclusion tends to increase slightly with age (rising from a DAI of 31.2 to 34.88), this difference was not statistically significant.

### 3.3. Emerging Digital Technologies for DHH Individuals

Several emerging technologies have been investigated as potential tools to improve communication and access to care for DHH individuals, including mobile health (m-health) applications, virtual and augmented reality (VR/AR), and artificial intelligence (AI)-based systems, such as machine learning approaches, vision-based technologies, wearable devices, and systems based on virtual avatars [19,71,72,73,74,75,76,77].

In particular, m-health and telemedicine solutions may potentially improve health education, patient-clinician interactions, and access to services and care, while VR and AR technologies may be used to facilitate communication, promote empathy, and reduce anxiety associated with medical procedures. AI-based systems, including machine learning approaches, vision-based technologies, wearable devices, and virtual avatars, might enable real-time recognition and translation of sign language and spoken language, potentially helping to increase communicative autonomy.

These technological innovations may likely shape a digital system aimed at promoting solutions for accessibility and inclusion in healthcare settings among DHH individuals.

#### 3.3.1. Mobile-Health Applications

In recent years, the rapid advancement of digital technologies has created new opportunities for improvement, including the mobile–health (m-health) applications which have emerged and are actually principally used as promising tools to support educational and behavioral interventions. This should be especially relevant for deaf and hard-of-hearing individuals, who often face communication barriers in traditional healthcare settings, including dental environments [6].

In this context, m-health applications offer an opportunity to enhance access to information, improve understanding of oral hygiene practices, and facilitate a more familiar and less anxiety-inducing introduction to the dental setting [6].

The use of m-health applications to enhance communication has been evaluated in the study of Lyall et al. [19], in which medical students and doctors were asked to transcribe a standardized set of sentences simulating a typical post-operative conversation with cochlear implant patients using three different methods: handwriting, computer typing, and smartphone application-based speech-to-text dictation (Dragon Dictation application). The time required for each method, as well as transcription accuracy, was systematically evaluated. The results demonstrated that m-health application dictation was significantly faster than both handwriting and computer typing, with a mean completion time of 17.8 s compared to 59.2 and 44 s, respectively. However, this increased speed was associated with a statistically lower accuracy, as speech-to-text transcription via m-health application achieved a mean accuracy of 92.5%, compared to 99.5% for handwriting and 99.8% for typing. Overall, while smartphone-based speech recognition significantly enhances communication speed, it may compromise accuracy, highlighting a trade-off that should be considered when using such tools in clinical interactions with DHH individuals.

Moreover, an m-health application system called DITE^TM^ (Deaf in Touch Everywhere) [71] has been proposed with the aim of improving communication between deaf and hard-of-hearing individuals and healthcare providers in Malaysia, a context where access to interpreting services is limited and often unregulated. The application was developed from the recognition that communication barriers in healthcare could lead to clinical errors, delays in treatment, poor treatment adherence, and, consequently, reduced access to healthcare services for the deaf population [71].

The application consisted of a digital telemedicine platform that allowed DHH users to quickly and securely connect with sign language interpreters via real-time video call systems [71]. The application allowed both advance booking and immediate requests for an interpreter, thereby overcoming the geographical and time limitations of traditional services [71]. In addition to video calls, the application included messaging features and the ability to share visual content, such as sign language GIFs, and health education materials, making communication more accessible for individuals with limited written language skills [71].

The application was developed using a participatory approach, directly involving both deaf individuals and interpreters, to tailor the features to the needs of users [71].

The application showed a high level of acceptance among the 18 study participants, since it was perceived as useful in facilitating access to interpreters, improving communication with healthcare professionals, and reducing the risk of misunderstandings with the deaf and hard-of-hearing population [71]. Furthermore, participants expressed a strong intention to use it in the future, noting that the availability of such a tool could increase their likelihood of seeking healthcare services, potentially promoting social inclusion and access to services [71].

However, among the limitations, technical issues related to the application’s navigation and speed emerged, along with the need to optimize the booking system and the management of interpreter availability, as well as issues regarding internet connectivity and privacy protection, which represent key elements for the future phases of the application’s development and implementation [71].

Furthermore, Convery et al. [78] reported another alternative approach to use m-health applications for remote healthcare. The study examined the effectiveness of an application called ReSound Assist™ designed for teleaudiology, which allowed patients to request and receive remote adjustments to their hearing aids [78]. The study, conducted on 30 adults, showed that asynchronous care via the application was as effective as in-person visits [78]. This approach may highlight how m-health applications could potentially facilitate not only communication but also ongoing clinical management, reducing the need for transportation and improving access to care in this population.

Recent evidence has explored the use of mobile applications to improve dental care for DHH individuals, adopting approaches focused on preventive education and clinical communication [72,73].

The Telesmile application was developed aimed at improving oral hygiene knowledge and practices among 25 Arab adolescents with hearing impairments (ages 12–18) [72]. Participants were divided into two groups: a control group receiving traditional instructions via booklets and an intervention group that used the application [72].

The Telesmile application was designed as a teledentistry platform, including educational videos in sign language with Arabic subtitles, specifically developed for DHH individuals. The content covered fundamental aspects of oral health literacy, including oral hygiene, brushing techniques, flossing, mouthwash use, diet, and regular check-ups [72]. Additionally, the app offered an interactive component that allowed patients to submit requests, describe symptoms, and upload images, facilitating communication with the dental professional [72].

The primary outcome was oral health knowledge, assessed using a validated 14-item questionnaire administered before and after 4 weeks of intervention. The use of the application showed a statistically significant improvement in oral health literacy [72].

In particular, the rate of correct responses regarding brushing frequency increased from 48% at baseline to 92% in the application group, compared with a modest increase in the control group (from 16% to 24%) [72]. Relevant improvements were also reported in other fields, such as knowledge of mouthwash use, knowledge of the frequency of dental visits, and the selection of toothbrushes, toothpaste, and proper oral hygiene techniques [72]. Overall, all questionnaire domains showed a significant increase in deaf individuals using the application compared to the conventional methods used in the control group, demonstrating the potential of the digital approach based on visual content and sign language for the deaf community [72].

Another approach was offered by the Odontoseñas application [73], which aims to improve communication during dental visits for deaf patients, enabling appointments without the need for interpreters. The study population consisted of adult deaf patients from the Chilean Deaf community, with varying degrees of hearing loss, including profound deafness [73].

The application was developed through an iterative co-design process involving dentists, deaf patients, and sign language interpreters, resulting in the creation of 13 videos in Chilean Sign [42] reported that in deaf/mute individuals. The content was organized according to the stages of the dental visit (identification, diagnosis, treatment, and post-operative instructions) and included standardized phrases related to symptoms, procedures, and clinical instructions [73].

The application’s usability and patient satisfaction during the visit were assessed using the System Usability Scale (SUS) and a modified version of the Dental Visit Satisfaction Scale (DVSS), respectively [73]. The results showed a high level of usability, with a SUS score of 96/100 [73]. Moreover, the impact on satisfaction among deaf patients showed that the average DVSS score increased from 21.3 to 29.3 out of 30 with the use of the app [73]. In patients with profound deafness, the effect is more pronounced, with an increase from 14.8 to 28.8, highlighting how the tool is particularly effective for individuals with greater communication difficulties [73]. Furthermore, 100% of participants reported satisfaction while using the application, underscoring the potential of the system’s acceptability in this community to enhance better communication [73].

#### 3.3.2. Virtual and Augmented Reality

Among emerging digital technologies, it has been suggested that Virtual Reality (VR) and Augmented Reality (AR) could play a supportive role for DHH individuals, serving as potential tools to overcome communication barriers and reduce stress and anxiety during clinical procedures [74,79,80,81,82].

In particular, VR and AR have increasingly been proposed as a strategy to improve doctor-patient communication among DHH individuals, potentially addressing one of the main challenges in healthcare for this population [79].

Indeed, immersive and digital technologies could potentially compensate for the limitations of the auditory channel through visual, multidimensional, and interactive means, potentially enabling more accessible and inclusive communication for this population [79,81].

The use of VR in enhancing communication has been more studied in simulation and educational settings, with the aim of improving healthcare professionals’ communication skills when interacting with this population [79]. In particular, VR has been proposed to create immersive environments and clinical simulations with standardized patients, in which professionals have to manage communicative interactions using visual signals, sign language, and interpreters, simulating realistic consultation conditions [79].

For instance, to promote empathy training in healthcare, immersive content based on 360° VR educational videos has been developed [83]. The content was designed using user-centered approaches that involved the DHH community in defining scenarios and communication needs, thereby potentially improving understanding of the real barriers patients experience [83]. Furthermore, VR has also been used to simulate hearing loss in medical students, allowing them to experience communication difficulties during clinical interactions and thereby potentially increase their empathy and awareness of these challenges, and foster better communication strategies when interacting with DHH individuals [80].

Moreover, VR was used for virtual simulation for training nurses for virtual clinical consultations mediated by interpreters, allowing healthcare professionals to practice communication skills, adjust consultation timelines, and interprofessional collaboration [84].

In addition to VR, AR has also been proposed as a potential digital technology to support communication in this population [82]. These approaches are based on integrating digital information into the real-world context and maximizing the visual channel, enabling more accessible and immediate communication for DHH individuals through AR glasses that can convert speech-to-text in real time, offering a potential solution to help deaf patients or those with severe hearing impairments follow a conversation [82]. However, the device’s performance appeared to be affected by ambient noise [82]. Nevertheless, compared to VR, the application of AR has not yet been validated or established in medical or dental settings, serving primarily as an extension of digital assistive technologies that could potentially facilitate communication.

Evidence has also highlighted the potential use of these tools in the context of clinical dental practice among this population to reduce stress or anxiety during clinical procedures [74,75].

For instance, the use of VR was investigated during professional oral hygiene combined with the Modified Tell-Show-Do (MTSD) technique, which incorporated sign language, in 40 pediatric subjects with severe to profound hearing loss, ranging from 6 to 11 years of age [74]. Compared to the group treated with the MTSD technique alone, the combination of VR and MTSD showed a relevant reduction in dental anxiety levels during the dental procedure [74]. Indeed, in the VR and MTSD group, pulse rate values were significantly lower, with an average reduction in heart rate of 6.95 ± 10.70 beats per minute (bpm), while an increase of 8.55 ± 11.11 bpm was observed in the MTSD alone group [74]. Moreover, the postoperative score on the Facial Image Scale (FIS) was 2.15 ± 0.75 in the VR group, compared with significantly increased scores in the MTSD group by 0.10 [74].

Similarly, a pilot study evaluated the effectiveness of visual distraction in managing dental anxiety through VR intervention during professional oral hygiene in 24 children with hearing and speech impairments (ages 6–12) [75]. The mean anxiety scores during and after treatment were significantly lower in the VR group (2.25 ± 0.89 and 1.13 ± 0.35, respectively) compared to both the no-distraction group (3.50 ± 1.31 and 1.88 ± 1.13) and the non-VR visual distraction group via tablet (2.88 ± 0.64 and 2.38 ± 0.92) [75]. However, no statistically significant differences were found in physiological parameters, including heart rate, among the groups [75].

By contrast, the use of VR during local anesthesia and pulpotomy was investigated in children aged 5–7 years. Compared to conventional methods of behavior control, the use of VR during the dental procedure did not show a relevant difference in anxiety levels, measured through Venham Clinical Anxiety Scale, heart rate, and modified Facial Affective Scale, potentially highlighting the limits of VR in more complex clinical procedures in this population.

#### 3.3.3. Artificial Intelligence, Machine Learning, Vision-Based Systems, Wearable Devices, and Virtual Avatars

Artificial intelligence (AI) is emerging as a potentially useful tool for reducing communication barriers among DHH individuals. Currently available solutions can be broadly classified into vision-based or audio-based systems, which utilize environmental inputs such as images, videos, or voice signals without requiring additional hardware, and device-based systems, which use wearable sensors or motion-tracking devices [76]. In this context, machine learning represents an innovative approach aimed at improving communicative autonomy and reducing dependence on human interpreters [85]. At the same time, the evolution of digital technologies has fostered the development of more advanced solutions, including virtual avatars and simulated environments capable of translating spoken language into sign language or vice versa, as well as integrated systems based on wearable devices and intelligent interfaces that enhance real-time communication accessibility [85]. These approaches form a hybrid technological interface where multiple systems may converge to promote inclusion, equity, and the active participation of DHH individuals.

Among vision-based systems, several deep learning models have been employed to potentially assist communication in different sign languages, including American, Turkish, Russian, and Arabic [86]. These models, through AI, were capable of extracting spatial features (such as hand shape and orientation) and temporal dynamics (gesture evolution over time) from video sequences of hand gestures that were processed and translated into textual outputs and, in some cases, into spoken language [86]. Most of these systems focused on gesture-to-text or gesture-to-audio translation, rather than full bidirectional communication [86]. The literature reports high accuracy levels of these systems, despite being affected by some limitations, such as lighting conditions, background complexity, and camera positioning, as well as by the difficulties in translating continuous sign language [86].

Among device-based systems, the use of wearable technologies has been reported, which allow movements to be captured directly via wearable sensors [87]. In these systems, artificial intelligence is used to process data from inertial sensors, accelerometers, and gyroscopes, with the aim of reconstructing hand and finger movements and translating them into linguistic output [87]. A system based on the integration of a smart ring and a smartwatch was able to recognize American Sign Language gestures with 94.2% accuracy across a vocabulary of approximately 100 words, maintaining high performance even as complexity increased [87].

Moreover, the development of increasingly complex integrated wearable systems was reported, such as the Enssat app [18], which combined mobile devices and head-mounted displays, such as Google Glass, to support real-time communication for DHH individuals. This system enabled the immediate transcription of spoken language, multilingual translation, and the detection of ambient sounds through visual notifications or vibrations, reducing dependence on human intermediaries and enhancing user autonomy [18]. In particular, the system utilized speech-to-text and machine translation services to convert speech into text displayed directly on the wearable device, with response times of less than approximately 2 s, positioning itself as a potential “real-time” solution, with the added capability of integrating ambient sound recognition functions, potentially increasing safety and awareness of the surrounding environment [18]. The system showed high levels of acceptance and satisfaction among users, potentially highlighting how these technologies can improve the quality of communication, despite presenting some limitations related to device battery life, environmental conditions, and the management of multiple conversations.

Nevertheless, these AI-based hybrid systems may represent a valuable solution to accurately support communication for DHH individuals in various contexts, including healthcare.

In addition to these approaches, evidence has reported the use of advanced technological systems for the automatic and real-time translation of text into sign language using 3D digital avatars, focusing specifically on Indonesian sign languages.

The system developed for the Indonesian language was a device-based system prototype, as it used motion capture sensors to track and record the human movements needed to build the gesture database [77].

Through artificial intelligence, the system used an m-application that was capable of translating text into animations, combining hand gestures and synchronized lip movements, generated via the Dirichlet Free-Form Deformation method [77]. A cross-fading algorithm was implemented to manage transitions between signs, ensuring natural transitions with fast response times of less than 100 milliseconds [77]. To evaluate the prototype, 10 participants were involved, comprising both sign language experts and potential users, who tested the usability using the SUS and its accuracy using the Mean Opinion Score (MOS) test [77]. The SUS score obtained was 76.25, placing the prototype in the “good” category and confirming that the interface was functional and well-received by users [77]. In terms of visual quality, the MOS test specifically for lip movements achieved a score of 4.422, indicating a very high perception of naturalness among the evaluators [77]. Furthermore, satisfaction surveys also showed that the animations generated by the system were considered very realistic [77].

Overall, AI-based systems may hold promising potential for improving communication in the healthcare setting, contributing to the development of solutions aimed at increasing accessibility and inclusion for DHH individuals.

Table 5 summarizes the characteristics of the included studies concerning emerging digital technologies for healthcare settings for DHH individuals.

## 4. Discussion and Barriers

Individuals with disabilities often require tailored healthcare services and specialized expertise [37,88]. Specifically regarding DHH individuals, the major barrier impeding equal healthcare access is effective communication with healthcare providers [13]. In the dental setting, multiple factors affect the oral health of DHH individuals, including the patient’s age, the severity of the impairment, the living environment, linguistic barriers, and the patient’s educational level, especially in medical and dental terminology [2,3,37].

In the modern digital era, technological advancements have increasingly permeated various sectors of healthcare [89]. Translating these advances to the oral health setting, which remains a particularly critical yet often overlooked domain, digital solutions may play a key role in enhancing communication, improving oral health literacy, and reducing access barriers for DHH individuals.

### 4.1. Oral Health and Disease Status in DHH Populations

Across the available evidence, DHH individuals appear to experience a less favorable oral health status than the general population, with a substantial burden of preventable oral diseases and unmet treatment needs [3,5,7,8,9,37,38,39].

Periodontal findings suggest a recurrent tendency toward poorer gingival and periodontal conditions. Although results were heterogeneous across studies, the comparison of PI, GI, and OHI-S indexes with those of other individuals with disabilities or healthy subjects [5,37,38,40,41,42,43,44,45,46,47,48,49,50,51,52,53,54,55] probably indicates that daily plaque control and access to preventive periodontal care may be suboptimal in many DHH patients.

Dental caries emerged as one of the most prevalent oral conditions across both primary and permanent dentitions [90]. In several studies, DHH individuals also showed worse caries indices than other study populations [2,3,4,7,9,10,20,37,38,39,54,56,57], reinforcing concerns regarding inequalities in oral healthcare access.

Beyond disease prevalence, malocclusions and orthodontic treatment needs were frequently reported. Moderate-to-severe occlusal alterations, crowding, increased overjet or overbite, open bite, spacing anomalies, and a relevant proportion of treatment need according to orthodontic indices suggest that developmental and functional oral conditions may represent an additional unmet area of care. Although severe skeletal discrepancies were not consistently observed, dental malocclusions were common enough to justify earlier screening and referral pathways [8,40,41,68,69,70].

Evidence on dental anomalies was more limited but still noteworthy, particularly in relation to agenesis, structural defects, fluorosis, and eruption disturbances [7,11,24,46]. Conversely, the near absence of data on oral mucosal conditions highlights an important research gap.

An important aspect to consider when interpreting these findings is the relatively young age range of the included populations (5–30 years), although no age-based restrictions were applied to the eligible population. This range encompasses critical developmental stages, from childhood to young adulthood, during which most oral diseases are largely preventable [91,92]. Furthermore, certain conditions, such as periodontitis or lesions of the oral mucosa, are less common in children and young adults, whilst others, such as gingivitis and dental caries, are more frequently observed even in this population [91,92]. The target population in terms of age is also reflected in the types of periodontal indices investigated in the included studies, in which indices indicative of gingival inflammation and plaque retention—such as GI, PI, and BoP—were extensively investigated, whereas indices such as PPD and CAL, which are more indicative of the destructive activity of periodontal disease, were less recorded [93].

However, the observed burden of plaque-related conditions, dental caries, and orthodontic treatment needs in DHH individuals is unlikely to be explained by age-related biological risk alone. Rather, it may reflect early and persistent barriers affecting oral health, including difficulties in implementing effective oral hygiene behaviors, limited access to preventive care, and reduced oral health literacy [21].

The consistency of these findings across different oral health domains suggests that disparities may emerge early and persist into adulthood, potentially leading to a cumulative burden of disease over time [94]. This highlights the importance of early preventive strategies targeting DHH children and adolescents, with continuity of care into adulthood [21].

Therefore, improving oral health in this population likely requires not only conventional preventive and therapeutic approaches but also inclusive models of care to address healthcare barriers [13].

### 4.2. Oral Health Awareness and Knowledge, Clinical Implications, and Digital Support Strategies in DHH Populations

The Simplified Oral Hygiene Index (OHI-S) in the present study showed a mean value of 1.97, indicating a moderate level of oral hygiene. This finding aligns with previous evidence suggesting that individuals with disabilities, particularly those who are DHH, often exhibit suboptimal oral hygiene conditions [2,3,4,7,37]. This condition has been partially attributed to limited oral health awareness and knowledge of effective oral hygiene practices, which represent a major barrier in maintaining daily oral care among DHH individuals [3,4].

Evidence from the literature consistently highlights substantial gaps in oral health knowledge among DHH individuals. Mustafa et al. [7] reported that about 69% of the DHH participants were unaware of proper toothbrushing techniques, including recommended frequency and duration. Nearly 65% had not received information on preventive dental treatments, while about 73% had never accessed oral health education materials.

Similarly, Hashemi et al. [39] found that participants’ oral hygiene was very poorly maintained, with only 11% brushing their teeth twice per day and 90% never having used floss.

In addition to this evidence, Khattak et al. [4] found comparable knowledge lacks among individuals with other impairments, especially in developing countries where oral health education is often lacking [7,95,96,97].

In the present study, when compared with other disability groups, the observed OHI-S value appears relatively lower than those reported in individuals with Down syndrome, cognitive impairment, blindness, autism, and cerebral palsy, as highlighted by Gaçe et al. [42], with similar trends also described by Goud et al. [44] and Reddy et al. [52]. This may suggest that, although oral hygiene and related knowledge remain suboptimal, DHH individuals could exhibit comparatively better outcomes than other disability groups, possibly due to differences in cognitive or motor limitations affecting daily oral care practices.

Given the potential knowledge gaps, which may reflect the oral health status of DHH individuals, emerging digital tools could represent a promising complementary strategy to traditional health education programs. Deafness encompasses several different conditions (Supplementary File S1), varying levels of literacy, individuals in highly diverse socio-educational contexts, as well as different age groups [1,25,98]. In this context, uniform and standardized interventions may not be sufficient, whereas tailored digital tools may offer a more flexible approach to the specific needs of the individual [6].

For instance, the Telesmile app, developed for DHH adolescents, showed a statistically significant improvement in oral health knowledge compared to conventional paper-based materials [72]. Specifically, the correct response among participants regarding brushing frequency increased from 48% to 92% in the group using the application, while the improvement in the control group was lower [72]. Similarly, progress was observed regarding knowledge of the use of toothpaste or mouthwash, the frequency of dental visits, and the appropriate choice of oral hygiene tools [72]. These findings suggest that, at least in some subgroups, dynamic, repeatable content structured according to more familiar communication codes may be superior to traditional educational methods.

Furthermore, these digital interventions could also have concrete clinical relevance, as gaining more accurate knowledge about oral hygiene practices, the regularity of dental checkups, and preventive behaviors is one of the main determinants of daily health behaviors and improves oral health-related quality of life [99]. From this perspective, the value of digital applications could extend beyond simply increasing theoretical knowledge, indirectly contributing to more favorable clinical outcomes and effective preventive care.

One aspect that is often overlooked is that not all DHH have the same level of text literacy [100]. For some individuals with congenital deafness or limited early language exposure, reading booklets, informational brochures, or written instructions may be less effective. In such cases, prevention programs relying primarily on text may potentially risk only partially addressing the actual educational need [85]. Indeed, applications designed with visual content, explanatory videos, practical demonstrations, and potentially sign language modules could, however, reach even those groups that benefit least from conventional information sources. In this context, differences in oral health literacy are closely linked to levels of basic literacy among DHH individuals. Although they may potentially help, emerging digital technologies should be seen as complementary tools rather than a single, standardized format for communication and information provision that works for all DHH individuals. Indeed, it is likely that digital educational content, such as that delivered through m-health applications, should comply with digital accessibility standards suitable for the different DHH individuals’ needs (e.g., intuitive icons, plain language, and customizable visual layouts tailored to individual needs), while also taking into account variations in sign language, disparities in reading comprehension, and different cognitive profiles.

Conventional oral health literacy models often rely on verbal counseling during the visit, standardized brochures, or general instructions that may not be frequently tailored [101]. In addition to being heavily dependent on the time available during the clinical visit and the patient’s ability to process information received in a single session, these strategies may have an episodic nature, as the educational message is generally provided only once or on sporadic occasions during dental visits, without a reinforcement system, repetition, or structured verification of understanding over time. DHH individuals may further encounter difficulties with conventional methods because of the discontinuity of the educational process and the inherent inability of conventional methods to adapt to more visual, repetitive, and customizable learning methods [102]. Therefore, digital tools, such as applications, may overcome these limitations.

During childhood, these tools could be integrated with conventional educational methods to improve oral health literacy. Schools, special education teachers, specialized educators, dentists, healthcare professionals, and caregivers remain central figures [103] by educating children on the importance of maintaining adequate oral hygiene and supporting proper home-based oral care routines [10]. Therefore, ensuring that both children and their caregivers receive appropriate guidance and education in oral health is important, particularly for populations with specific clinical care needs [3,10].

In this context, the analysis of caries indices (in the present review, the mean DMFT was 1.97 and dmft 1.61) indicates that caries experience is already established across both permanent and primary dentitions in DHH individuals, highlighting a continuity of disease burden from childhood into adulthood. Notably, the predominance of the Decayed domain in both indices suggests the presence of untreated caries, reflecting limited awareness of oral health and disease. Moreover, when extending the analysis to surface-based indices, a similar pattern emerges (mean DMFS was 6.22 and dmfs 9.54), indicating a more extensive involvement of tooth surfaces. Moreover, the higher dmfs compared with DMFS values may also reflect the increased vulnerability of primary teeth and the cumulative effect of inadequate oral hygiene practices during early childhood [90,104].

Mobile apps could serve as a home-based extension of the educational messages received at school or during dental visits, ensuring continuity between the school environment and family routines [105,106]. In this perspective, digital technology could serve as an educational bridge for DHH individuals between dental professionals, teachers, and families, reinforcing the consistency of preventive messages [90,104].

Another critical element concerns the role of caregivers, which is particularly important not only among the pediatric population but also potentially significant for individuals with multiple disabilities or limited independence. Caregivers, in fact, not only assist with daily oral hygiene practices but also act as intermediaries between the healthcare system and the patient, directly influencing adherence to preventive and therapeutic regimens [107,108,109]. In this regard, digital technologies could also offer shared monitoring tools, with the potential to further strengthen the continuity of care and the stability of oral health behaviors over time [107,108,109].

In the adult population, the concern may not only be acquiring basic knowledge of oral health, but also maintaining preventive behaviors over time and adhering to regular dental checkups [108,110]. In this perspective, digital reminders, brief educational modules, monitoring of home care routines, and follow-up provided by digital tools such as m-health applications could promote greater continuity of care, especially among patients who tend to visit the dentist only when symptoms are already manifest [111]. Indeed, the experience reported of the ReSound Assist app, which showed efficacy comparable to in-person visits in the remote management of hearing aids, suggests that digital models may also be well-accepted among the DHH adult individuals [78]. Although developed in the field of audiology, this example may be adapted to dentistry for preventive reminders. In fact, taken together, the parallel interpretation of DMFT/dmft and DMFS/dmfs indices suggests that not only the prevalence but also the extent and severity of dental caries remain significant concerns in this population.

However, the heterogeneity of the DHH population may also suggest the need for differentiated approaches. For instance, in individuals with syndromic deafness or deafness associated with cognitive disabilities, highly simplified, icon-based content, also including caregivers, may be more appropriate. In children or adolescents, interactive and gamified models may prove more effective [112]. For adults and elderly with acquired hearing loss, tools focused on maintaining routines and secondary prevention with a simple interface design and considering family support may be more effective [113,114].

The ability to tailor content to the patient’s profile is one of the main advantages of digital systems over conventional educational tools, which are inherently more static in both content and form [6,115].

However, for most of the digital interventions used for the DHH population, no classification distinction was made between the different types of deafness (congenital, prelingual, acquired, or associated with complex syndromes), nor between the different levels of language exposure and communication skills. Consequently, one of the potential interpretive limitations concerns the generalization of results to an extremely heterogeneous population, in which educational and support needs can vary considerably. However, digital applications could represent particularly promising tools because they are potentially adaptable also to the broader functional, cognitive, and socio-educational needs of the individual.

### 4.3. Dental Care Access, Clinical Implications, and Digital Support Strategies in DHH Populations

Accessibility can be conceptualized as the result of the interaction between the availability of healthcare services, often shaped by environmental barriers, and individuals’ ability to effectively access them [22]. Patients should undergo dental visits at least semiannually for routine check-ups and treatments as needed. Furthermore, certain patients including subjects with special needs, may benefit from more frequent follow-up at 2, 3, or 4 months [3,9,10,116].

Within this framework, DHH individuals experience reduced access to healthcare [117] primarily because of the communication barrier, but also due to economic constraints, organizational inefficiencies, perceived inconvenience, fear, and challenges in establishing an effective patient–provider relationship [7,9]. Collectively, these barriers contribute to healthcare disparities and inadequate health literacy within the deaf community [2,117].

Although the underlying causes remain not fully elucidated, this disparity appears to be multifactorial, involving sociodemographic, educational, and cultural factors, as well as limited prioritization of impairment-oriented healthcare services [22].

Consistent with these findings, several studies have reported that a substantial percentage of individuals with disabilities have never visited a dentist in the past, or only access dental appointments irregularly [4]. This pattern of underutilization further contributes to the progression of untreated oral conditions and reinforces oral health inequalities.

This trend is reflected in the results of the present comprehensive review regarding caries prevalence, which generally increased with age, and the Filled domain often accounted for a smaller proportion of the DFMT [38,45,60,66]. This pattern may plausibly reflect the underutilization of restorative dental services.

In pediatric populations, particularly among children with multiple disabilities, such as in the syndromic form of deafness (Supplementary File S1), reduced access to dental care may be further influenced by caregiver-related factors. These include the relatively low priority often assigned to oral health, as well as limited awareness within families regarding the importance of preventive oral hygiene practices [5,9,10,37]. Moreover, because many oral conditions are perceived as non-life-threatening and easily treatable, preventive dental care is often deprioritized, leading to delayed care-seeking behaviors [22].

A significant correlation was found between class II dental malocclusions and concomitant systemic conditions [8]. Moreover, evidence on dental anomalies was more limited but still noteworthy, particularly in relation to agenesis, structural defects, fluorosis, and eruption disturbances. These findings may be especially relevant in syndromic or genetic forms of hearing impairment, where multiple dental anomalies are more frequent, oral manifestations can coexist with systemic features, and require multidisciplinary management [118].

Dental anxiety represents an additional barrier that can contribute to reduced access to dental care, especially in pediatric populations [119,120]. This may contribute not only to avoidance behaviors in patients but also to reluctance among caregivers to seek dental care. Furthermore, low health literacy may exacerbate dental fear, resulting in unmet treatment needs [10,11,12]. However, in this comprehensive review, only one study [67] analyzed the dental fear and anxiety of DHH individuals, recording no statistically significant correlation with DMFS or the number of Decayed, Missing, or Filled surfaces.

VR and AR may also enhance patient understanding and reduce anxiety [74,79]. In particular, VR-based interventions combined with adapted communication strategies (e.g., sign-supported Tell-Show-Do techniques) have been associated with reduced anxiety in pediatric DHH patients [74] potentially facilitating healthcare accessibility. However, their effectiveness appears to vary depending on the complexity of the treatment [75] suggesting that these tools should be considered as complementary rather than primary strategies.

Organizational barriers also play a role in limiting access to dental services. Previous evidence has shown that DHH individuals frequently encounter difficulties in scheduling appointments, with requests often denied due to the perceived need for interpreter services [21,117]. Specifically, Schniedewind et al. [117] reported that dental clinics were more than six times as likely to deny new patient requests due to interpreter-related factors compared with primary care clinics. Patients have also reported repeated unsuccessful attempts to contact clinics, including unanswered calls or interrupted communication [117].

The impact of these barriers extends beyond clinical indices. A significant positive correlation was reported between the Pediatric Oral Health-Related Quality of Life and DMFT, dmft, PI, and GI, suggesting that poorer oral health status, characterized by higher caries experience, plaque accumulation, and gingival inflammation, is directly associated with a worsening perceived quality of life in children [43]. Within this framework, improving access to dental care and promoting early preventive strategies may contribute not only to reducing disease burden but also to enhancing quality of life in DHH individuals.

Within this context, emerging digital technologies may represent an important opportunity to improve accessibility to dental care for DHH individuals [19,71,72,77]. In particular, m-health applications, telemedicine platforms, and AI-based communication systems may facilitate initial contact with dental services, appointment scheduling, and pre-visit communication, potentially reducing missed appointments [16,115].

In particular, m-health applications such as Telesmile and Odontoseñas demonstrate how digital tools can enhance access to dental care by improving communication before and during appointments [72,73]. These systems reduce reliance on intermediaries by providing sign language-based educational and clinical content, enabling patients to better understand appointment procedures and treatment plans [72,73]. Notably, high usability and satisfaction scores suggest that such tools may improve patient engagement and reduce appointment avoidance behaviors [72,73]. However, while they may simplify access to clinical care, these digital solutions should be considered supplementary, complementary support tools rather than automatic substitutes for personalized solutions and human support. For some DHH individuals, barriers such as the digital divide, high device costs, and unequal access to high-speed Internet connectivity may limit widespread adoption, potentially exacerbating health care disparities among DHH individuals with socioeconomic disadvantages.

Interestingly, across all technological categories, a common theme emerges: digital tools may primarily enhance access to dental care by facilitating communication at multiple stages of care [72,73]. After all, in the DHH individuals, oral health disparities appear more closely linked to the communication barrier, which uniquely restricts their access to preventive education and effective clinical dialogue [21]. In fact, the DHH individuals does not necessarily lack the physical or cognitive capacity for self-care, while primarily imposing limitations on oral hygiene maintenance in other special health care need groups.

The evidence highlighted that the clinical oral status of DHH individuals presents a heterogeneous pattern when compared to other disability groups. While several studies reported higher caries indices and poorer periodontal health in DHH subjects compared to visually impaired individuals [41,44,45,52,62] or healthy controls [58,59], a more consistent trend emerged regarding cognitive and neuromotor disabilities. Specifically, DHH individuals generally exhibited lower caries experience and better oral hygiene than individuals with Down syndrome, intellectual disabilities, or cerebral palsy [39,42,60,61,63].

### 4.4. Dentist-Patient Communication, Clinical Implications, and Digital Support Strategies in DHH Populations

Studies have shown that individuals with disabilities frequently experience communication challenges, increased anxiety, and sensory sensitivities that make dental treatments difficult to perform [4].

Within this context, DHH individuals represent a particularly vulnerable population due to the communication challenge. In particular, children with hearing impairment often face significant challenges in expressing their needs and difficulties to both clinicians and caregivers, as they primarily rely on sign language and lip-reading for communication [10].

For this reason, based on the age of onset (Supplementary File S1), once identified, hearing loss should be appropriately managed to minimize its adverse effects and to ensure that subjects with hearing impairment remain as independent as possible in everyday activities, including healthcare-related behaviors [1]. In contrast, the absence of effective communication tools or trained healthcare providers may significantly compromise the quality of care [117] potentially leading to misdiagnosis and misinterpretation of DHH patients’ health conditions, thus contributing to the development of feelings of frustration, fear, mistrust and the avoidance of healthcare treatments [21,117]. Collectively, these factors undermine the development of a strong patient–clinician relationship [21,117].

From the clinicians’ perspective, communication barriers were also reflected in their perceived level of confidence. Most of the dentists participating in the study by Korleski et al. [121] stated being more comfortable treating DHH patients who communicated orally than those using American Sign Language (ASL), a finding useful for planning educational courses. Among the DHH individuals, orally DHH individuals are subjects educated to communicate primarily through spoken language, with sign language used as a supplementary communication modality [8]. As a consequence, ASL users face even more difficulties in receiving both healthcare and dental services, and their condition is worsened if they have a lower education level [21].

In a clinical setting, to overcome this communication barrier, patients were frequently asked to bring a family member to their appointments to act as an interpreter [22,117]. The presence of an interpreter has ensured greater adherence to recommendations; otherwise, patients often fail to understand the diagnosis and discontinue treatment [117]. The lack of professional ASL interpreters and the lack of use of sign language by healthcare providers represent, unfortunately, a common barrier to healthcare communication [2,22,117]. In the case of ASL users, clinicians were usually not able to understand ASL without the presence of an interpreter, which is necessary to ensure a proper level of communication [117]. For example, Miranda et al. [2] reported that 56% of Brazilian dentists struggle with DHH communication, with 98% needing interpreters. However, the literature indicates that, in several cases, even when patients informed healthcare staff about their hearing loss, no accommodation was implemented to facilitate communication, or interpreting services were often promised but ultimately not provided [117].

In line with these considerations, the difference in oral health outcomes highlighted in the present comprehensive review across different groups of DHH individuals stratified based on severity classification (Supplementary File S1) may reflect variability in communication difficulties associated with different levels of hearing loss.

In fact, a positive correlation between the severity of hearing loss and DMFS values was found, suggesting that individuals with more severe impairment may experience a greater cumulative burden of caries at the surface level [67]. However, subgroup analyses showed that older adolescents with profound hearing loss had lower DMFS values compared with younger children with moderate or severe hearing impairment [67]. This apparent inconsistency may be explained by differences in communication modalities and adaptation: individuals with profound hearing loss, particularly at older ages, are more likely to rely on structured and consistent communication systems (e.g., sign language) and may develop more effective compensatory strategies, facilitating clearer interactions with caregivers and healthcare providers [122]. Conversely, individuals with moderate hearing loss often depend on partial verbal communication, which may lead to misunderstandings, less effective transmission of oral health instructions, and reduced adherence to preventive behaviors [122]. These differences in communication effectiveness may ultimately influence oral health awareness and knowledge. These findings illustrate how severity of hearing loss and preferred communication modality directly influence both caries burden and the type of digital support that is most appropriate.

Beyond caries outcomes, in orally DHH individuals, the registered high prevalence of malocclusions and orthodontic needs may be partially explained by the tendency of orally deaf individuals to move their jaws more during speech and by the differences in tongue movement trajectories compared to hearing people, as it is known that impaired function of the maxillofacial muscles is a contributing factor in bite pathology [8]. During physiological rest, DHH children may experience an increase in masticatory muscle activity, which could result in changes to occlusion morphology or complicate current orthodontic problems [8].

Limiting to the severity of hearing loss, Sokolohorska-Nykina et al. [8] reported no significant correlation with the type of malocclusion, suggesting that functional or behavioral factors, such as the type of communication, may play a more relevant role.

In this context, telemedicine-based applications enabling real-time access to sign language interpreters, such as video-call platforms, may overcome the common barrier of a lack of an interpreter [71,117]. Among the available communication modalities, the video relay service (VRS) is one of the most commonly used in healthcare. This telecommunication system enables DHH individuals to communicate in ASL via videophone, with an interpreter translating the signed message into spoken language for the hearing party [117].

These systems allow both scheduled and on-demand communication, reducing logistical constraints and potentially increasing healthcare utilization among DHH patients. However, technical limitations, including internet connectivity, system usability, and data privacy concerns, remain critical factors influencing their implementation [71]. Therefore, in this context, clinical implementation of digital support strategies for DHH individuals to enhance dentist-patient communication, such as telemedicine and digital communication tools, should address critical issues related to patient privacy, data protection, and regulatory compliance when handling sensitive diagnostic information.

Emerging AI-based technologies may offer alternative solutions aimed at reducing reliance on informal interpreters. Vision-based systems and wearable devices have shown promising accuracy in translating sign language into text or speech [76,87], while integrated systems combining mobile devices and smart wearables enable real-time speech-to-text conversion and environmental awareness [18]. These approaches may enhance communicative autonomy; however, their performance is still influenced by environmental factors such as lighting conditions, background noise, and device positioning [76].

Moreover, advanced systems based on virtual avatars capable of translating text into sign language have demonstrated good usability and high perceived realism [77]. These tools may be particularly useful for standardized clinical communication, such as delivering instructions or explaining procedures, although their application in complex, bidirectional interactions remains limited [77].

Speech-to-text transcription or writing back and forth, where feasible, may represent another effective strategy to clarify potential misunderstandings during bi-directional clinical interactions, especially in post-lingual DHH individuals (Supplementary File S1) [21,121]. Many DHH individuals rely on lip-reading to understand clinicians; however, because healthcare providers often speak too quickly, this approach may be unreliable and lead to misunderstandings [22]. Therefore, promoting the acquisition of sign language skills among healthcare professionals may represent a more effective and accurate strategy [22].

Speech-to-text applications have been shown to significantly reduce communication time compared to traditional methods such as handwriting or typing, although at the expense of slightly reduced transcription accuracy [19]. This trade-off suggests that, while these tools may enhance efficiency, their use should be carefully considered in contexts requiring high precision, such as diagnosis explanation or informed consent.

Overall, the findings of the present review suggest that digital technologies should not be considered as standalone solutions but rather as part of a multimodal communication approach. The integration of traditional methods (e.g., interpreters, sign language skills) with digital tools may represent the most effective strategy to improve accessibility and quality of care for DFF individuals.

Interestingly, variability in communication effectiveness across different degrees of hearing loss may also influence the impact of digital technologies. Individuals with profound hearing loss, who are more likely to rely on structured visual communication systems such as sign language, may benefit more from technologies specifically designed around visual interfaces (e.g., sign language-based applications or avatar systems) [73]. Conversely, individuals with moderate hearing loss, who often rely on partial verbal communication, may experience greater benefit from speech-to-text and hybrid communication tools [122].

From a clinical perspective, the integration of these technologies into dental practice requires not only technical implementation but also adequate training of healthcare professionals and consideration of patient-specific communication needs [9].

However, interestingly, some patients have reported relatively better communication experiences in clinical contexts requiring fewer explanations, although this may reflect reduced interaction rather than effective communication [13]. This finding suggests that apparent improvements in communication may sometimes mask a lack of meaningful exchange, potentially limiting patient understanding and engagement in care.

### 4.5. Socio-Cultural Barriers, Clinical Implications, and Digital Support Strategies in DHH Populations

Another aspect to consider is social marginalization, which often results in discrimination against DHH individuals. In this context, “ableism” refers to the belief that specific physical and cognitive characteristics are prerequisites for a life of value [12,123]. This perspective shapes societal norms and influences how variations in physical or mental functioning, whether congenital or acquired—are perceived [12,124,125].

While the term “hearing impaired” is frequently employed in clinical settings, it may evoke negative reactions within the DHH community; many individuals do not view hearing loss as an impairment but rather as a defining characteristic of a linguistic minority [23].

Ableist attitudes contribute to social prejudice, discrimination, and structural oppression, which may also be reflected in legislation, public policies, and healthcare practices. These factors, in turn, may partially explain the reduced utilization of dental care services among individuals with disabilities [12,123].

These socio-cultural differences are further compounded by intersectionality. Donald et al. [21] report that DHH individuals from marginalized racial/ethnic groups, with lower education or ASL use, face severely underserved dental needs, including linguistic/cultural barriers, low oral health literacy, financial constraints, reduced preventive care, and higher periodontitis rates among the United States, black and latinx populations.

In the same study, DHH women were more likely to report unmet dental needs, and lower communication scores were significantly associated with lower educational attainment and self-identification with marginalized groups [21]. Additionally, financial hardship emerged as a major barrier to accessing oral healthcare, particularly among women with lower educational levels [21]. Consistently, broader evidence suggests that economic constraints significantly hinder dental care utilization and oral health outcomes, with younger adults being especially vulnerable to financial barriers [21].

In line with this evidence, Vyavahare et al. [58] reported a statistically significant association between DMFT scores and socioeconomic status among DHH individuals, whereas Jain et al. [45] did not find significant associations with caste or level of education.

Within this socio-structural framework, it is interesting to note that a large part of the studies included in the present comprehensive review were set in India (19/34, 56%) [5,9,38,41,44,45,46,47,48,50,52,53,58,61,64,65], as also documented previously in a scoping review [124]. This pattern may reflect socio-structural influences rather than epidemiological differences. In fact, research in this field is frequently based on cross-sectional designs and institutionalized populations, which are more readily accessible in certain settings, such as specialized schools for DHH students [125]. Furthermore, the strong focus on oral health inequalities and vulnerable groups in countries such as India, together with documented unmet oral health needs among individuals with disabilities, may contribute to the higher research output observed in these contexts [125].

Interestingly, this geographical distribution is also reflected in the development of digital and technological solutions aimed at improving dental care for DHH individuals. The studies included in this review that proposed specific technological tools in the dental field were predominantly conducted in India [74,75], with additional contributions from Saudi Arabia [72] and Chile [73]. This pattern suggests that innovation in this area may be driven not only by technological advancement but also by the need to address existing healthcare needs [6,126]. However, at the same time, the limited geographical interest highlights a gap in the global implementation of digital support strategies, particularly in high-income settings where such tools could be more widely integrated into routine dental care [6,126].

### 4.6. Dentistry Training, Clinical Implications, and Digital Support Strategies in DHH Populations

Limited dental training was reported as another significant barrier to the provision of dental care for DHH individuals. Evidence indicates that dentists are generally less likely to treat patients with special healthcare needs [11], with a substantial proportion reporting that they do not feel adequately qualified to manage this population [22,39,121].

Consistently, Korleski et al. [121] reported that fewer than one in seven dentists considered their undergraduate dental education appropriate for treating DHH patients, and only a minority had participated in continuing education (CE) courses on the topic. However, the same study also identified a strong interest among practitioners in further education, with many respondents expressing willingness to attend CE courses and to involve their staff [121]. Notably, more recent graduates tended to report more positive perceptions of their educational experience, suggesting a gradual improvement in curricular exposure [121].

Dentists with greater knowledge of disabilities and prior clinical experience with patients with special healthcare needs tend to report fewer perceived barriers and an increased likelihood of providing care to this population [88,121,123].

Thus, designing continuing education courses on the topic may be an important instrument in educating dental care providers about treating these patients [121]. In this respect, many universities already offer courses for undergraduate students aimed at increasing future dentists’ willingness to treat patients with special health care needs [11]. Indeed, one of the most relevant educational aspects of the emerging technologies presently reported is the potential to foster empathy and perspective-taking among healthcare providers [127]. For instance, VR has been used in medical training to simulate hearing loss during clinical consultations, allowing learners to directly experience the frustration and communication difficulties associated with incomplete or hard-to-understand messages [80]. In dentistry, such an approach could increase the professional’s awareness of the barriers that often might be unaddressed in theoretical courses but profoundly influence the patient experience as well as the providers’ cultural competence, which is frequently deemed insufficient and a source of discouragement in the effective management of this patient population [6,128]. In this context, it may be worth considering that training programs should prepare dental professionals not only to use emerging assistive technologies, but also to recognize their technical and ethical limitations. Training programs should make clear that digital devices, while promising in many respects, cannot replace human empathy and interpersonal relationships, and should reinforce clinicians’ skills in data confidentiality, emphasizing that, in many cases, they require coordinated, multidisciplinary collaboration with certified professional interpreters alongside digital aids.

Conversely, the lack of training may have significant clinical implications in the oral management of DHH patients, who often require specific care. For example, in the present comprehensive review, over half of the orthodontic subjects (53.49%) presented a DAI score ≥ 26, indicating a need for treatment, with more than one in four falling into the “mandatory treatment” category. Moreover, Sokolohorska-Nykina et al. [8] reported that the severity of malocclusion tends to increase slightly with age, although this difference was not statistically significant. This trend across age groups may potentially suggest that the lack of proper training may contribute to unmet dental needs [128].

Beyond formal education, limited familiarity with available support resources further constrains access to care [110,129]. Dentists, particularly those working in smaller practices, may be unaware of the procedures required to accommodate DHH patients, including how to access interpreting and communication services [21]. This highlights the need for improving both the availability and the accessibility of such resources, especially for professionals with limited prior exposure to this population [21].

Importantly, targeted educational interventions have demonstrated measurable benefits. A longitudinal study conducted in Jamaica reported a significant improvement in access to dental care for DHH patients following the introduction of a mandatory sign language course for predoctoral dental students [121].

In this context, emerging digital technologies could represent a valuable evolution of traditional training models. Conventional training often relies heavily on lectures and theoretical content, which may limit learning, while interactive virtual environments may offer a more immersive experience [130]. These digital tools enable learners to develop technical skills while also gaining a firsthand understanding of the relational, organizational, and emotional challenges patients face throughout their treatment process [80,127].

Such training technologies could be particularly relevant because they enable the practice of skills that might be rarely taught within traditional training programs, including managing visit timing, maintaining eye contact, nonverbal communication, adapting the clinical setting, and designing a fully inclusive environment. This factor is particularly noteworthy considering that the challenges encountered by DHH subjects do not arise solely during the clinical procedure, but can begin well before the actual therapeutic session, affecting the preliminary stages of the care pathway [117].

Virtual simulation environments could also be used for analyzing and improving internal organizational processes. By reconstructing the entire patient journey, from scheduling an appointment to check-in at the practice and receiving care, these technologies could enable the testing of procedures, the preparation of administrative and front-office staff, the identification of critical steps, and the implementation of corrective solutions [130].

**Figure 3:**
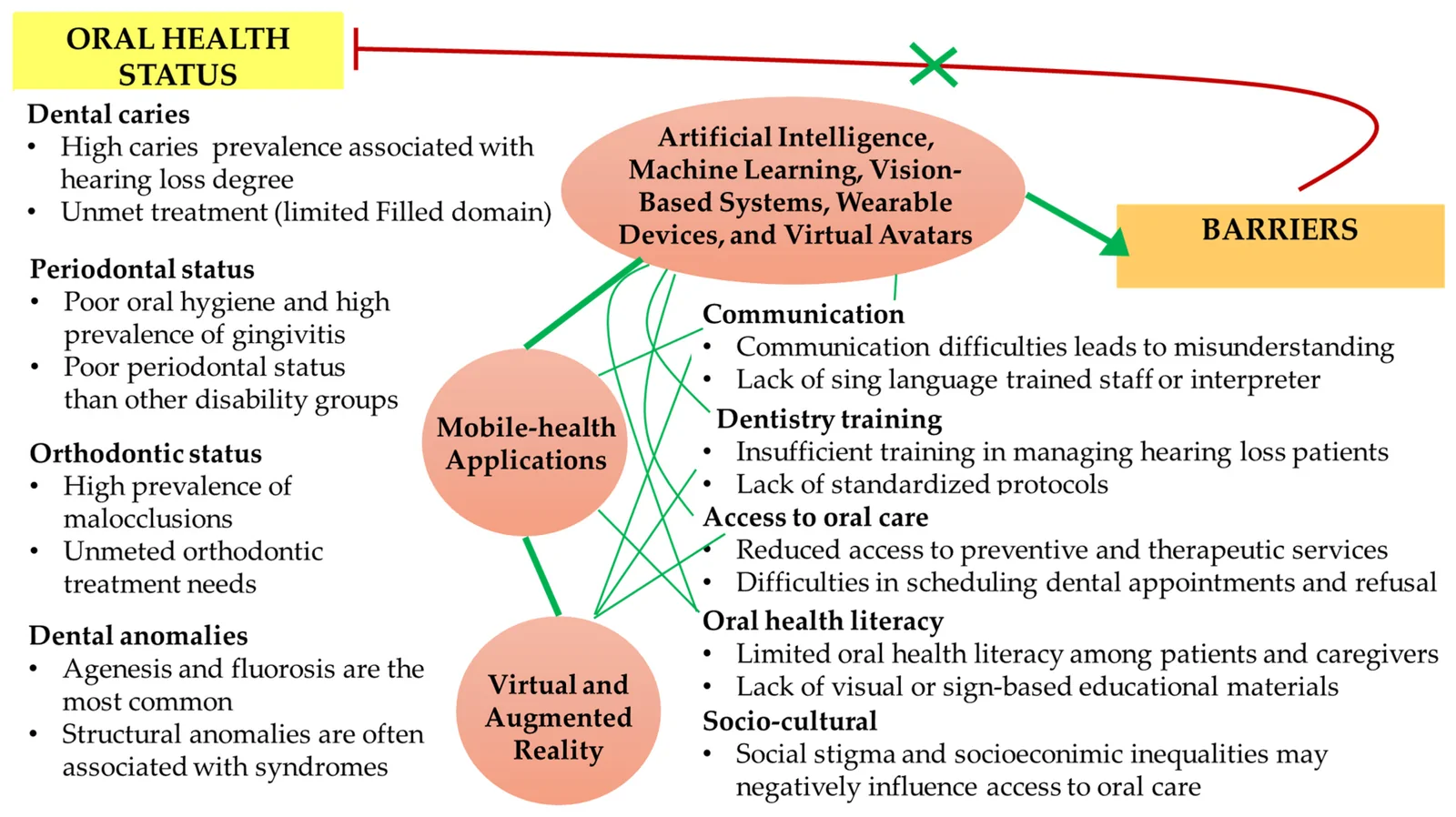
Schematic overview of the main findings related to the oral health status of deaf and hard-of-hearing individuals, the principal barriers affecting access to and delivery of oral healthcare (red lines with cross), and the potential influences of emerging technologies in overcoming these barriers (green lines).

Figure 3 summarizes the main findings related to the oral health status of deaf and hard-of-hearing individuals, the principal barriers, and the potential influence of emerging technologies.

### 4.7. Limitations, Strengths, and Future Prospects

Despite the insights provided, several limitations of this comprehensive review should be acknowledged.

First, the narrative nature of the study may introduce bias compared to a systematic approach, as a formal quantitative risk of bias assessment was not performed. Furthermore, significant methodological heterogeneity exists among the included studies, particularly regarding the study designs, different indices used to measure caries and periodontal status, and the frequent reliance on small sample sizes, predominantly from school-based or institutional convenience samples. Consequently, the reported summary estimates and pooled mean indices should be interpreted with caution because of underlying clinical heterogeneity in age ranges and diagnostic criteria across cohorts.

Furthermore, the available literature focuses predominantly on pediatric and young adult populations (age range: 5–30 years), leaving the oral health needs of the DHH elderly individuals underexplored.

The generalizability of these findings may also be limited by the geographical concentration of the included studies in Asian countries; consequently, the reported prevalence of dental caries, oral hygiene status, periodontal disease, and malocclusions may be influenced by specific regional, cultural, or socio-economic factors.

Additionally, many of the digital solutions discussed remain in experimental phases and lack large-scale validation in diverse clinical settings.

Nevertheless, this comprehensive review possesses distinct strengths, offering a comprehensive and multidisciplinary perspective that bridges the gap between clinical oral health data and the specific communicative challenges faced by the DHH individuals. By distinguishing between different degrees of hearing loss, ages of onset, and linguistic preferences, such as sign language versus oral communication. Moreover, the study provides a structured framework that highlights the critical interplay between systemic barriers, oral health outcomes, and the potential of digital aids, moving beyond descriptive analysis to offer practical directions for addressing health disparities.

Looking ahead, future perspectives should prioritize the development of tailored digital tools based on the specific type of DHH classification, due to the different needs for congenital or post-lingual impairments for example.

Furthermore, longitudinal controlled trials should be conducted to evaluate the long-term impact of m-health and immersive technologies on objective clinical outcomes like DMFT and plaque indices. Moreover, professional training for dental healthcare providers should be updated to include technological assistance as a core competency, aiming to strengthen the therapeutic alliance.

Ultimately, the goal should be a shift from isolated technological tools to integrated, inclusive care systems that ensure health equity for the deaf community in the digital era.

## 5. Conclusions

DHH individuals appear to experience substantial oral health disparities, characterized by a higher burden of periodontal diseases, untreated dental caries, and unaddressed orthodontic needs. The findings of the present comprehensive review seem to suggest that these inequalities do not stem from intrinsic biological or cognitive limitations, but rather from persistent communication barriers, reduced oral health literacy, and limited professional training among healthcare providers. However, the magnitude and direction of these disparities vary across studies, populations, and settings.

In this context, emerging digital technologies, including mobile-health applications, artificial intelligence-based translation tools, 3D sign language avatars, and virtual reality, may offer promising solutions to bridge the communication gap, alleviate procedural anxiety, and facilitate preventive oral care. However, these factors should be regarded as potential explanatory tools rather than well-established digital support already available in clinical practice due to the heterogeneity and the often preliminary nature of the current evidence.

Furthermore, transitioning from isolated technological tools to an integrated, inclusive oral healthcare framework, comprising continuing education in special care dentistry, is essential to guarantee health equity, patient autonomy, and improved quality of life for the deaf community in the digital era.

To optimize clinical translation, further studies are needed to evaluate the long-term clinical efficacy of these technologies while explicitly accounting for the clinical heterogeneity within the DHH population.

## Figures and Tables

**Table 1:** Periodontal status in DHH individuals. Abbreviations: Deaf and Hard-of-Hearing (DHH), plaque index (PI), gingival index (GI), bleeding on probing (BoP), periodontal probing depth (PPD), clinical attachment level (CAL), simplified oral health index (OHI-S), community periodontal index (CPI).

Study	Country	DHH Population Characteristics	Periodontal Status and Indices	Main Results and Statistically Significant Findings
Al-Maweri et al., 2015 J Clin Pediatr Dent [37]	Yemen	Sample size: 50 Age: 9.84 ± 2.26, range 6–14 years Target population: DHH students of 8 special needs schools in Sanaa; control with other special needs students at the same schools	PI: 1.19 ± 0.54 GI: 1.13 ± 0.60	The mean PI was lower compared with subjects blind (1.62 ± 0.54), with compound disabilities (1.61 ± 0.71), cognitive impairments (1.41 ± 0.60), or physically impaired (1.26 ± 0.49). The mean GI was lower compared with subjects blind (1.84 ± 0.79), with compound disabilities (1.63 ± 0.62), cognitive impairments (1.42 ± 0.65), or physical impairments (1.28 ± 0.60).
Alyami et al., 2022 Cureus [40]	Saudi Arabia	Sample size: 116 Age: 5–16 years Target population: DHH children in Jeddah city; no control	Gingivitis: 74	In about 63.79% of DHH children, a gingivitis status was retrieved.
Avasthi et al., 2011 Int J Dent Clin [41]	India	Sample size: 264 Age: range 5–16 years Target population: DHH students at special needs schools in Delhi and Gurgaon; control with other special needs students at the same schools	Gingivitis was detected in 131 (49.65%) subjects.	The prevalence of gingivitis in DHH individuals was lower compared with that of the blind group (71.53%).
Gaçe et al., 2014 Mater Sociomed [42]	Albania	Sample size: 147 Age: median age 13.0 years Target population: DHH students at special needs schools in Birmingham; control with other special needs students at the same schools	OHI-S: 2.42	The mean OHI-S score showed statistically significant differences when compared among the different disability groups. The mean OHI-S score in deaf-mute individuals was higher than that of individuals with Down syndrome (1.99), cognitive impairment (1.96), blindness (1.62), autism (1.27), and cerebral palsy (1.05).
Ghannam et al., 2024 BDJ Open [43]	Syria	Sample size: 90 Age in the male subgroup: 9.7 ± 0.25 Age in female subgroup: 9.6 ± 0.31 Target population: DHH students at the Institute of Special Education for Hearing Impaired in Syria; no control	PI in the male subgroup: 1.9 ± 0.81 PI in the female subgroup: 1.5 ± 0.09 GI in the male subgroup: 0.9 ± 0.09 GI in the female subgroup: 1.1 ± 0.14	Males showed a higher PI, indicating major plaque buildup, while females recorded a higher GI, suggesting more gingivitis than males. A significant positive correlation was found between the Pediatric Oral Health-Related Quality of Life and PI and GI.
Goud et al., 2021 J Family Med Prim Care [44]	India	Sample size: 207 Age: range 6–24 years Target population: DHH students at the special needs schools in the district of Gulbarga; control with other special needs students at other special needs schools	OHI-S: 2.43 Periodontal health (CPI 0): 22 (16.9%) BoP was recorded in 4 (3.1%) subjects (CPI 1) Calculus was retrieved in 102 (78.5%) subjects (CPI 2) PPD (4–5 mm) was retrieved in 2 (1.5%) subjects (CPI 3) PPD ≥ 6 mm was never retrieved (CPI 4)	DHH children had a statistically significantly higher frequency of healthy periodontium compared with visually impaired children (4.4%). The OHI-S was higher in DHH children compared with visually impaired children (1.95).
Jain et al., 2008 J Oral Sci [45]	India	Sample size: 127 Age: range 5–22 years Target population: DHH students in a school for DHH in Udaipur City.	Periodontal health (CPI 0): 72 (24.2%) BoP was recorded in 63 (21.2%) subjects (CPI 1) Calculus was retrieved in 33 (11.1%) subjects (CPI 2) PPD (4–5 mm) was retrieved in 105 (35.4%) subjects (CPI 3) PPD ≥ 6 mm was retrieved in 24 (8.1%) subjects (CPI 4)	CPI scores were worse in DHH individuals compared with those of blind individuals, who had a higher frequency of CPI 0 (43.4%) and lower in CPI 3 (28.4%) and CPI 4 (6.0%). No statistically significant differences were found regarding CPI scores between males and females.
Jnaneswar et al., 2017 J Indian Soc Pedod Prev Dent [46]	India	Sample size: 540 Age: range 5–15 years Target population: institutionalized DHH children in Khordha district; no control	Periodontal health (CPI 0): 156 (28.9%) BoP was recorded in 129 (23.9%) subjects (CPI 1) Calculus was retrieved in 255 (47.2%) subjects (CPI 2)	No statistically significant differences were found regarding CPI scores between males and females.
Kondepudi et al., 2024 J Indian Soc Pedod Prev Dent [47]	India	Sample size: 72 Age: 13.5 ± 3.55 Target population: DHH students residing in special care schools in Nellore district; no control	PI: 0.28 ± 0.38 GI: 0.13 ± 0.19	Improved oral hygiene knowledge was registered after an educational program for DHH individuals through video skits and sign language.
Kumar et al., 2008 Spec Care Dentist [48]	India	Sample size: 127 for the OHI-S index and 86 for the CPI index Age: range 5–23 years Target population: DHH students at the boys’ school for DHH in Udaipur; no control	OHI-S: 1.86 ± 0.85 Periodontal health (CPI 0): 36 (42%) BoP was recorded in 31 (36%) subjects (CPI 1) Calculus and PPD (4–5 mm) were retrieved in 19 (22%) subjects (CPI 2 and 3) PPD ≥ 6 mm was never retrieved (CPI 4)	No statistically significant difference was found between age subgroups for the OHI-S index. CPI 1 (bleeding) was higher in the 12–17 subgroup compared with the 18–23 subgroup, while CPI 2 and CPI 3 were lower.
Moin et al., 2024 Glob Pediatr Health [49]	Pakistan	Sample size: 60 Age: range 7–20 years Target population: DHH of 12 special needs schools in Pakistan; no control	OHI-S: 3.05 ± 2.15 GI: 1.19 ± 0.54	Skit video and pictorial intervention significantly improved OHI-S and GI compared with sign language.
Rawlani et al., 2010 J Kor Dent Sci [50]	India	Sample size: 137 Age: 7–18 years Target population: DHH of the school for DHH in Warora; no control	OHI-S: 1.49 ± 0.76 CPI: 0.46 ± 0.31 PI: 0.81 ± 1.46 CAL: 0.26 ± 0.15	The OHI-S and CAL indices were statistically significantly higher among females compared to males. No statistical differences were found between females and males concerning the PI and CPI indices.
Rezaei et al., 2019 J Family Med Prim Care [51]	Iran	Sample size: 37 Age: 9.9 ± 1.70, range 6–12 years Target population: DHH of schools in Kermanshah; control with blind of the same study	PI: 0.87 ± 0.14 GI: 1.39 ± 0.30	Both PI and GI were slightly higher among DHH individuals compared with those of the blind (0.81 ± 0.19 and 1.35 ± 0.32, respectively).
Reddy et al., 2013 J Indian Soc Pedod Prev Dent [52]	India	Sample size: 95 Age: range 7–17 years Target population: DHH children attending institutionalized visually impaired and hearing impaired in Bhopal city; control with visually impaired	OHI-S: 1.15 ± 0.72	The OHI-S index of DHH individuals was significantly better compared with that of visually impaired individuals (1.51 ± 0.93).
Samal et al., 2024 Cureus [53]	India	Sample size: 91 Age: 15.45 ± 2.51 Target population: DHH students of 10 schools; no control	Gingival bleeding was detected in 82.42% of subjects	Statistically significant difference in gingival bleeding was found in relation to age, but not with gender or the severity of hearing impairment.
Sandeep et al., 2016 Indian J Dent Res [5]	India	Sample size: 180 Age: 10.83 ± 2.9 years Target population: DHH students at the special school for DHH in Bhimavaram Town	PI: 1.70 ± 0.61 GI: 1.59 ± 0.58	About 81% of deaf and hard-of-hearing individuals had moderate-abundant deposits of plaque, while about 78% moderate-severe gingival inflammation.
Shaw et al., 1986 Community Dent Oral Epidemiol [54]	United Kingdom	Sample size: 240 Age: 11 years Target population: DHH students at special needs schools in Birmingham; control with other special needs students at the same schools	Calculus was detected in 23% of subjects	When compared to the other disability groups, calculus was detected in the DHH individuals at a lower frequency compared with the epilepsy (42%), visually impaired (38%), heart disease (33%), blood disorders group (28%), cognitive impairment (28%), physically impaired (26%), and higher than those with maladjusted (22%), speech or language impaired (21%) and autism (9%).
Shivakumar et al., 2017 J Int Oral Health [38]	India	Sample size: 150 Age: range 5–22 years Target population: DHH students at a school for the sensory impaired in Satara district; no control	OHI-S: 1.51 ± 0.85 PI: 0.79 ± 1.32 CAL: 0.32 ± 0.19 CPI: 0.53 ± 0.41	The OHI-S and CAL index were statistically significantly higher in males than in females. The PI and CPI index did not show statically significant difference between males and females.
Tefera et al., 2022 Clin Cosmet Investig Dent [55]	Ethiopia	Sample size: 149 Age: 15.51 ± 3.904, range 7–30 years Target population: DHH students at special needs schools in Birmingham; control with other special needs students at the same schools	The prevalence of periodontal disease was 22.8% BoP was detected in 43.6% of subjects	In about 29.2% of DHH individuals above the age of 18, periodontal disease was diagnosed.

**Table 2:** Dental caries in DHH individuals. Abbreviations: Deaf and Hard-of-Hearing (DHH), Decayed, Missing, and Filled Teeth (DMFT/dmft); Decayed, Extracted due to caries, Filled Teeth (DEFT/deft); Decayed, Missing, and Filled Surface (DMFS/dmfs).

Study	Country	DHH Population Characteristics	Caries Index	Main Results and Statistically Significant Findings
Al-Maweri et al., 2015 J Clin Pediatr Dent [37]	Yemen	Sample size: 50 Age: 9.84 ± 2.26, range 6–14 years Target population: DHH students of 8 special needs schools in Sanaa; control with other special needs students at the same schools	DMFT: 1.91 ± 2.07 dmft: 4.37 ± 3.11 DMFS: 2.91 ± 3.78 dmfs: 9.40 ± 9.40	The mean DMFT was lower compared with subjects with compound disabilities (2.85 ± 1.98) or cognitive impairment subjects (2.37 ± 2.59), but higher compared with blind (1.44 ± 1.56) or physically impaired (2.37 ± 2.59). The mean dmft was lower compared with physically impaired subjects (4.68 ± 3.30), but higher compared with subjects with compound disabilities (4.28 ± 2.91), cognitive impairments (4.27 ± 3.12), or blind (3.44 ± 3.1). The mean DMFS was lower compared with subjects with compound disabilities (4.81 ± 4.36) or cognitive impairment subjects (4.75 ± 7.05), but higher compared with blind (2.23 ± 2.99) or physically impaired (1.36 ± 2.48). The mean dmfs were lower compared with physically impaired subjects (10.10 ± 9.26) or cognitive impairments (9.71 ± 10.18), but higher compared with subjects with compound disabilities (8.22 ± 7.54), or blind (5.78 ± 5.64).
Aruna et al., 2005 J Indian Assoc Public Health Dent [65]	India	Sample size: 200 Age: 6–18 years Target population: DHH students at the Residential School of Davangere; no control	DMFT: 1.64	The mean DMFT was slightly higher in the subgroup with up to 11 years (1.181) compared with the 12–15 subgroup (1.65) and the 16–18 subgroup (1.46).
Alyami et al., 2022 Cureus [40]	Saudi Arabia	Sample size: 116 Age: 5–16 years Target population: DHH children in Jeddah city; no control	Among 116 DHH children, 25 had decayed, 12 had missing, and 30 had filled teeth.	About 25.8% of DHH children had undergone filling treatment, 10.3% had missing teeth, and 21.5% had decayed teeth.
Avasthi et al., 2011 Int J Dent Clin [41]	India	Sample size: 264 Age: range 5–16 years Target population: DHH students at special needs schools in Delhi and Gurgaon; control with other special needs students at the same schools	Caries were detected in 72.43% of subjects, while 27.57% were caries-free	The prevalence of dental caries in DHH individuals was higher compared with that of the blind group (59.68%).
Chand et al., 2014 J Clin Diagn Res [61]	India	Sample size: 155 Age: 3–22 years Target population: DHH students of 4 special needs schools in Indore city; control with other special needs students at the same schools	Caries were detected in 45.8% of subjects, while 54.2% were caries-free DMFT: 1.10 ± 1.58 deft: 0.85 ± 1.76	The mean DMFT score of the DHH individuals was slightly higher than that of subjects with visual impairment (0.97 ± 1.46). However, this score was lower than that of subjects with cerebral palsy (1.22 ± 1.89), and significantly lower than that of subjects with cognitive impairment (2.06 ± 2.36). The DHH individuals recorded the highest mean deft score among all disability groups, including the cognitive impairment (0.16 ± 0.75), visually impaired (0.46 ± 1.19), and cerebral palsy (0.53 ± 2.84).
Gaçe et al., 2014 Mater Sociomed [42]	Albania	Sample size: 147 Age: median age 13.0 years Target population: DHH students at 9 special needs schools of Albania; control with other special needs students at the same schools	DMFT: 4.7 ± 3.9 deft: 2.8 ± 2.9	When compared with other disability groups, the mean DMFT score in deaf-mute individuals was lower than that of individuals with cognitive impairment (5.8 ± 5.2) and cerebral palsy (5.6 ± 8.1), but similar to that of blind individuals (4.7 ± 3.9) and higher than that of individuals with autism (2.6 ± 3.2). In the primary dentition, the mean deft in deaf-mute individuals was lower than in cerebral palsy (4.5 ± 4.2), and blind individuals (4.4 ± 4.2), but higher than in Down syndrome (1.9 ± 2.7)
Ghannam et al., 2024 BDJ Open [43]	Syria	Sample size: 90 Age in the male subgroup: 9.7 ± 0.25 Age in female subgroup: 9.6 ± 0.31 Target population: DHH students at the Institute of Special Education for Hearing Impaired in Syria; no control	Caries were detected in 93% of subjects, while 7% were caries-free DMFT in the male subgroup: 2.5 ± 0.25 DMFT in the female subgroup: 2.3 ± 0.33 dmft in the male subgroup: 4.1 ± 0.49 dmft in the female subgroup: 3.3 ± 0.52	The DMFT and dmft scores were similar between the female and male subgroups. A significant positive correlation was found between the Pediatric Oral Health-Related Quality of Life and DMFT/dmft.
Goud et al., 2021 J Family Med Prim Care [44]	India	Sample size: 207 Age: range 6–24 years Target population: DHH students at the special needs schools in the district of Gulbarga; control with other special needs students at other special needs schools	DMFT: 1.24 Decayed domain: 1.24 Missing domain: 0 Filled domain: 0 dmft: 0.36 decayed domain: 0.36 missing domain: 0 filled domain: 0	The mean DMFT score of DHH individuals was slightly higher than the reported mean score of visually impaired individuals (0.98), while the dmft scores were lower (0.52 in visually impaired).
Hashemi et al., 2012 J Oral Health Oral Epidemiol [39]	Iran	Sample size: 21 Age: 14.7 ± 7 years Target population: DHH students of 3 special needs schools and 2 special needs care home; control with other special needs students at the same schools and care home	DMFT: 4.95 ± 2.10	The mean DMFT score of DHH individuals was slightly higher than the reported mean score for other disability groups, including subjects with physical impairments (4.67 ± 3.18), and other unspecified disabilities (4.88 ± 2.93), while were lower than subjects with cognitive impairments (5.26 ± 3.49), and with Down Syndrome (8.67 ± 3.51). However, no statistically significant difference across disability groups was found.
Jain et al., 2008 J Oral Sci [45]	India	Sample size: 127 Age: range 5–22 years Target population: DHH students in a school for DHH in Udaipur City.	DMFT: 2.61 Decayed domain: 2.3 Missing domain: 0.19 Filled domain: 0.15 dmft: 0.83 ± 1.60 DMFS: 3.69 Decayed domain: 2.69 Missing domain: 0.88 Filled domain: 0.18 dmfs: 1.40 ± 2.81	An increased prevalence with age in dental caries was found. Adults had a higher number of decayed teeth. The Decayed domain was the main statistically significant contributor to the total DMFT index. Missing and Filled surfaces were not accounted for a major proportion in any age subgroup with the exception of the 18–22 age group. For the dmft and dmfs the 5–7 age subgroup showed the significantly highest values. DMFT was associated with age, but not with caste, socioeconomic status, level of education, or degree of hearing loss.
Jain et al., 2013 Oral Health Dent Manag [66]	India	Sample size: 297 Age: range 4–23 years Target population: DHH students of institutionalized hearing-impaired and blind students in Udaipur; control with blind students of the same schools	DMFT: 1.97 ± 1.93 Decayed Domain: 1.68 ± 1.73 Missing Domain: 0.20 ± 0.49 Filled Domain: 0.09 ± 0.29 dmft: 0.26 ± 0.85 decayed domain: 0.23 ± 0.76 filled domain: 0.02 ± 0.14	The Decayed domain was the main contributor to the total DMFT. The mean DMFT score for DHH individuals was statistically significantly higher than the reported mean score for other disability groups, including blind subjects (1.48 ± 1.29). The mean dmft score of DHH individuals was comparable to that observed in other disability groups, including blind subjects (0.28 ± 0.75), with no statistically significant difference.
Jnaneswar et al., 2017 J Indian Soc Pedod Prev Dent [46]	India	Sample size: 540 Age: range 5–15 years Target population: institutionalized DHH children in Khordha district; no control	DMFT: 0.37 ± 0.88 Decayed Domain: 0.29 ± 0.70 Missing Domain: 0.02 ± 0.17 Filled Domain: 0.06 ± 0.29 DEFT: 0.15 ± 0.56 Decayed domain: 0.12 ± 0.43 Extracted domain: 0.03 ± 0.20	A statistically significant difference was retrieved for the missing domain and DMFT, with lower values in the 5–10 years subgroup than in the 11–15 years subgroup. A statistically significant difference was retrieved for the decayed and extracted domains, and DEFT, with higher values in the 5–10 years subgroup than in the 11–15 years subgroup.
Kondepudi et al., 2024 J Indian Soc Pedod Prev Dent [47]	India	Sample size: 72 Age: 13.5 ± 3.55 Target population: DHH students residing in special care schools in Nellore district; no control	Caries were detected in 44.4% of subjects, while 55.6% were caries-free DMFT/dmft: 1.18 ± 2.11 DMFS/dmfs: 3.54 ± 6.93	Improved oral hygiene knowledge was registered after an educational program for DHH individuals through video skits and sign language.
Mishra et al., 2021 J Pharm Bioallied Sci [64]	India	Sample size: 267 Age: range 6–15 years Target population: DHH students at a special needs school in India; control with other special needs students at the same school	DMFT: 0.66 ± 1.30 dmft: 0.38 ± 1.06	The mean DMFT score of DHH individuals was lower than the reported mean score for other disability groups, including subjects with blind (1.48 ± 1.61), with multiple impairments (1.20 ± 1.58), and orthopedic impairments (0.81 ± 1.27). The mean dmft score of DHH individuals was higher than the reported mean score for other disability groups, including subjects with blind (0.36 ± 1.04), with multiple impairments (0.32 ± 1.05), and orthopedic impairments (0.27 ± 0.90).
Nqcobo et al., 2012 SADJ [63]	South Africa	Sample size: 99 Age: 9.2 ± 4.34 Target population: DHH students of special needs schools in Johannesburg; control with other special needs students of the same school	DMFT: 0.67 dmft: 3.03	The mean DMFT was the slightly lower compared with other disabilities, such as cerebral palsy (0.68), learning disability (1.20), and mental disability (1.27). The mean dmft were the lower compared with cerebral palsy (3.40), but higher than learning disability (1.35), and mental disability (2.83).
Rezaei et al., 2019 J Family Med Prim Care [51]	Iran	Sample size: 37 Age: 9.9 ±1.70, range 6–12 years Target population: DHH of schools in Kermanshah; control with blind of the same study	DMFT: 1.81 ± 2.16 Decayed domain: 1.49 ± 2.09 Missing domain: 0.22 ± 0.48 Filled domain: 0.11 ± 0.52 dmft: 2.08 ± 3.48 decayed domain: 2.03 ± 3.50 missing domain: 0.03 ± 0.16 filled domain: 0.03 ± 0.16	Among DHH individuals, high rates of dental caries have been reported, particularly in the primary dentition, with a higher prevalence of decayed domain in both deciduous and permanent dentition, potentially indicating limited access to dental care. Furthermore, a high unmet treatment need (0.85 ± 0.31) was found.
Rawlani et al., 2010 J Kor Dent Sci [50]	India	Sample size: 137 Age: 7–18 years Target population: DHH of the school for DHH in Warora; no control	DMFT: 2.53 ± 1.72 Decayed domain: 2.46 ± 1.57 Missing domain: 1.20 ± 0.45 Filled domain: 0.00	No statistically significant difference in DMFT was found regarding gender.
Reddy et al., 2013 J Indian Soc Pedod Prev Dent [52]	India	Sample size: 95 Age: range 7–17 years Target population: DHH children attending institutionalized visually impaired and hearing impaired in Bhopal city; control with visually impaired	DMFT: 1.4 ± 1.95 Decayed domain: 1.38 ± 1.93 Missing domain: 0.02 Filled domain: 0.00 dmft: 0.47 ± 1.01 decayed domain: 0.34 ± 0.77 missing domain: 0.14 ± 0.37 filled domain: 0.00	The mean DMFT score of DHH individuals was higher compared with the score of visually impaired individuals (0.94 ± 1.45). However, the difference was not statistically significant. The mean dmft score of DHH individuals was higher compared with the score of visually impaired individuals (0.19 ± 0.79). This difference was statistically significant.
Samal et al., 2024 Cureus [53]	India	Sample size: 91 Age: 15.45 ± 2.51 Target population: DHH students of 10 schools; no control	Caries were detected in 76.92% of subjects, while 23.08% were caries-free	Individuals with a worsened level of hearing loss recorded a significantly higher prevalence of dental caries. Age and gender were not significantly associated with dental caries prevalence.
Sandeep et al., 2016 Indian J Dent Res [5]	India	Sample size: 180 Age: 10.83 ± 2.9 years Target population: DHH students of the special school for DHH in Bhimavaram Town	Caries were detected in 65% of subjects, while 35% were caries-free DMFT: 1.9 ± 1.3 Decayed Domain: 1.8 ± 1.2 Missed Domain: 0.10 ± 0.39 Filled Domain: 0.07 ± 0.4 dmft: 2.0 ± 1.8 decayed domain: 1.5 ± 1.7 missing domain: 0.4 ± 0.8 filled domain: 0.07 ± 0.4	The Decayed domain was the highly statistically significant domain to the total DMFT and dmft; in the latter, the missed component was also statistically significant. The mean DMFT increased with age, specifically: 1.6 ± 1.3 (in 6–8 years old group); 1.9 ± 1.2 (in 9–12 years old group); 2.2 ± 1.2 (13–16 years old group). The mean dmft values distribution among age groups was: 2.8 ± 2.2 (in 6–8 years old group); 2.1 ± 1.5 (in 9–12 years old group); 1.1 ± 1.3 (13–16 years old group). A statistically significant difference was reported among age groups, specifically: between the 9–12 years old and 13–16 years old groups, and between the 6–8 years old group and 13–16 years old group. No statistically significant difference was reported between groups 6–8 and 13–16 years old.
Sanjay et al., 2014 J Int Oral Health [62]	India	Sample size: 195 Age: 6–20 years Target population: DHH students at the Institutes (Sensory impaired & Blind) in India; control with blind students at the same school	DMFT: 1.80 ± 1.264 Decayed domain: 1.64 ± 0.866 Missing domain: 0.14 ± 0.643 Filled domain: 0.02 ± 0.016 dmft: 0.33 ± 0.242 decayed domain: 0.33 ± 0.242 missing domain: 0.00 filled domain: 0.00	The mean DMFT in the DHH individuals was statistically significantly lower compared with that of blind individuals (2.16 ± 2.005). The mean dmft in the DHH individuals was statistically significantly higher compared with that of blind individuals (0.25 ± 0.265). In relation to age, there was a significant increase in the DMFT for the advancing age subgroup, while the dmft was higher among 6–10 years subgroup compared to the older age subgroup.
Shaw et al., 1986 Community Dent Oral Epidemiol [54]	United Kingdom	Sample size: 240 Age: 11 years Target population: DHH students at special needs schools in Birmingham; control with other special needs students at the same schools	DMFT: 1.76	The Filled domain was the main contributor to the total DMFT index. When compared to the other disability groups, the DHH individuals showed lower DMFT than the epilepsy (2.16) group, but higher than those with autism (1.26). In contrast, the speech or language-impaired subgroup showed one of the lowest DMFT indices (1.06) across the disability groups.
Shivakumar et al., 2017 J Int Oral Health [38]	India	Sample size: 150 Age: range 5–22 years Target population: DHH students at special needs schools in Birmingham; control with other special needs students at the same schools	DMFT: 2.89 ± 1.91 Decayed domain: 2.71 ± 1.92 Missed domain: 0.24 ± 0.83 Filled domain: 0.11 ± 0.95 DMFT in the 5–8 years old subgroup: 0.51 ± 0.80 DMFT in the 9–12 years old subgroup: 1.73 ± 1.69 DMFT in the 13–18 years old subgroup: 2.79 ± 1.98 DMFT in the 19–22 years old subgroup: 4.53 ± 2.39 DMFS: 3.91 ± 2.12	Analyzing the data by age groups, a statistically significant increase in mean scores was found, rising from the 5–8 years old group to the 9–12, 13–18, and reaching the 19–22 years old group. The Decayed domain was the main statistically significant contributor to the total DMFT index, in particular when compared to the other domains. The analysis of lesion severity through the DMFS showed that each affected tooth often presented multiple compromised surfaces.
Shyama et al., 2001 Community Dent Health [60]	Kuwait	Sample size: 312 Age: 13.2 years Target population: DHH students at special needs schools in Kuwait; control with other special needs students at the same schools	DMFT: 5.0 Decayed domain: 4.3 dmft: 5.3 decayed domain: 4.5 DMFS: 9 Decayed domain: 6.9 dmfs: 14.7 decayed domain: 11.2	When compared to the other disability groups included, the mean DMFT and DMFS of DHH individuals were significantly higher than those of the visually impaired group, and second only to the subjects with Down Syndrome. The decayed domain was the main contributor to the total index, both for the DMFT and the dmft.
Suma et al., 2011 Int J Clin Pediatr Dent [9]	India	Sample size: 76 (hearing and speech impairment) Age: range 5–18 years Target population: DHH students at the RV Integrated School for the Disabled in India	Caries were detected in 32 subjects, while 44 were caries-free	No statistically significant difference was reported in the gender distribution of the caries disease.
Tefera et al., 2022 Clin Cosmet Investig Dent [55]	Ethiopia	Sample size: 149 Age: 15.51 ± 3.904, range 7–30 years Target population: DHH students at special needs schools in Amhara Region; no control	Caries were detected in 38.9% of subjects, while 61.1% were caries-free DMFT: 1.15 ± 1.654 Decayed domain: 0.77 ± 1.258 Filled domain: 0.00 dmft: 0.11 ± 0.564	Dental caries were significantly associated with grade level, caregivers’ support or tooth brushing, oral health status, or medication intake.
Trimeridou et al., 2025 Eur Arch Paediatr Dent [67]	Greece	Sample size: 83 Age: 33 subjects with a mean of about 11 years; 50 subjects with a mean of about 16 years Target population: DHH students in elementary and high schools for DHH in Greece; no control	DMFT in 11-year subgroup: 1.9 ± 2.4 dmft in 11-year subgroup: 1.0 ± 1.9 DMFT in 16-year subgroup: 3.8 ± 3.9 DMFS in 11-year subgroup: 3.45 ± 5.4 (8.2 ± 8.3 for moderate and 8.75 ± 7.2 for severe hearing loss) dmfs in 11-year subgroup: 2.5 ± 5.2 DMFS in 16-year subgroup: 7.3 ± 9.0 (2.9 ± 3.3 for profound hearing loss)	A positive correlation was found between the severity of hearing impairment and the DMFS. High school students (16-year subgroup) with profound hearing loss had lower DMFS compared to children (11-year subgroup) with severe or moderate hearing loss. No statistically significant correlation was retrieved between gender or age and DMFS. Decayed, Missing, or Filled surfaces domains were not correlated with age, gender, or severity of hearing impairment. No statistically significant correlation was found between dental fear and anxiety and DMFS or the number of Decayed, Missing, or Filled surfaces.
Vyavahare et al., 2024 Bioinformation [58]	India	Sample size: 60 Age: 13.58 ± 1.139 Target population: DHH students of 2 schools for hearing and speech impaired children in Belagavi city; control with healthy children from another school	Caries were detected in 83.3% of subjects, while 16.7% were caries-free. DMFT: 6.80 ± 6002	The number of DHH children caries-free was statistically higher compared with healthy children caries-free (51.2%). The mean DMFT score was statistically significantly higher among DHH and speech-impaired individuals compared with healthy individuals (1.26 ± 1.719). No association between DMFT score and gender was retrieved, while it was recorded in relation to socioeconomic status.
Wei et al., 2012 Res Dev Disabil [59]	China	Sample size: 229 Age: 17–19 years Target population: DHH students at a special senior high school for DHH in Jiangsu; control with healthy adolescents of a normal senior high school	Caries were detected in 55.9% of subjects, while 44.1% were caries-free. DMFT: 1.40 ± 1.89 Decayed domain: 1.07 ± 1.62 Missing domain: 0.10 ± 0.39 Filled domain: 0.12 ± 0.46	The prevalence of dental caries in DHH individuals was statistically significantly higher compared with healthy individuals (13.8%). No difference in relation to gender was recorded. The DMFT index of DHH individuals was higher compared with healthy individuals (1.36 ± 1.72), but the difference was not statistically significant. Only the Missing and Filled index was statistically significantly higher in DHH individuals compared with healthy (0.04 ± 0.19 and 0.03 ± 0.20).

**Table 5:** Digital technologies for healthcare settings for DHH individuals. Abbreviations: Deaf and Hard-of-Hearing (DHH), missing data (MD).

Study	Design, Country and Aim	Technologies	Population Characteristics	Outcome
Alkhalifa et al., 2018 Multimed Tools Appl [18]	Design: prototype development Country: Saudi Arabia Aim: develop and evaluate Enssat, a mobile application integrated with Google Glass to provide real-time speech transcription, translation, and alerts for surrounding sounds	Type: assistive technology and mobile application using a head-mounted display Validation status: no formal clinical validation reported Clinical readiness: prototype Comparator: 15 individuals without hearing impairments	Sample size: 10 DHH individuals Target population: DHH university students Age: 20–25 years Language/sign-language context: Arabic- and English-speaking	System responsiveness and user acceptance
Al Khuzayem et al., 2024 Sensors [86]	Design: prototype development Country: Saudi Arabia Aim: develop Efhamni, an application for automatic translation of isolated Saudi sign language into Arabic text and audio to facilitate communication between DHH and hearing individuals.	Type: Android application developed using a deep-learning model Validation status: technical evaluation Clinical readiness: prototype; not yet suitable for clinical use Comparator: none	Sample size: 110 DHH individuals and 10 Saudi sign language participants for questionnaires; 3 DHH and 3 hearing individuals for usability testing Target population: MD Age: MD Language/sign-language context: Saudi and Arabic sign language	Usability, model performance (test accuracy, validation accuracy, precision, recall), and processing time
Al-Obodi et al., 2020 IJERT [76]	Design: development and technical validation study Country: Saudi Arabia Aim: develop an artificial intelligence-based tool for the recognition of Saudi Arabic sign language	Type: artificial intelligence-based sign language recognition system integrated into mobile and desktop applications Validation status: technical validation; no formal clinical validation reported Clinical readiness: prototype; not clinically evaluated Comparator: previously published sign language recognition models	Sample size: MD Target population: DHH individuals in varied environmental conditions Age: MA Language/sign-language context: Saudi Arabic sign language	Training accuracy
Blok et al., 2023 Clin Simul Nurs [83]	Design: feasibility and usability study Country: United States of America Aim: develop and assess an educational virtual reality video design to improve healthcare professionals’ skills when caring for DHH individuals	Type: virtual reality video using headsets Validation status: educational content was iteratively reviewed and refined by an advisory panel and other stakeholders Clinical readiness: educational innovation ready for use; the videos were implemented in the course and continued to be used in undergraduate courses Comparator: none	Sample size: 22 Target population: advanced practice nursing students Age: MD Language/sign-language context: American sign language	Feasibility, usability, qualitative feedback
Campos et al., 2020 Univers Access Inf Soc [73]	Design: feasibility and usability study Country: Chile Aim: develop and iteratively evaluate a sing language based mobile app to facilitate dentist-DHH patient communication	Type: Android/iPhone mobile app with Chilean sign language videos, subtitles, and dental communication content Validation status: no formal clinical validation reported Clinical readiness: Clinically evaluated prototype/final release version Comparator: dental examination and diagnosis explanation without the app	Sample size: 9 DHH individuals and 9 dental students/dentists in final simulations; additional groups of 4 DHH individuals and 3 dentists involved in earlier evaluations Target population: DHH individuals attending primary public healthcare dental facilities Age: MD Language/sing-language: Chilean sign language	App usability; dental-visit satisfaction; qualitative perceptions, comments, and suggestions from DHH individuals and dentists
Chaitanya et al., 2024 Int J Clin Pediatr Dent [74]	Design: randomized controlled trial Country: India Aim: establish the effectiveness of virtual reality eyewear in reducing dental fear and anxiety in DHH children	Type: virtual reality eyewear Validation status: no formal clinical validation reported Clinical readiness: clinically evaluated Comparator: no virtual reality eyewear	Sample size: 40 Target population: DHH children who need oral prophylaxis Age: range 6–11 years; mean 8 years Language/sign-language context: MD	Dental fear and anxiety, pressure rate
Chong et al., 2024 Digital Health [71]	Design: cross-sectional study Country: Malaysia Aim: establish feasibility, acceptability, and input to improve the design of the DITE^TM^ among DHH individuals and sign language interpreters	Type: DITE^TM^ app; Android smartphone; WhatsApp for participant support and communication; Zoom for virtual focus groups Validation status: no formal app validation reported Clinical readiness: feasibility evaluation, not clinically validated Comparator: none	Sample size: 18 Target population: 9 DHH individuals and 9 sign language interpreters Age: 25–50 years Language/sign-language context: Malaysian sign language	Feasibility, acceptability, user experience, perceived usefulness/ease of use, and feedback for app development; qualitative experiences
Convery et al., 2020 Telemed J E Health [78]	Design: feasibility study Country: Australia and the United States of America Aim: assess the usability of the ReSound Assist app enabling remote communication between patients and doctors	Type: app-based teleaudiology/remote hearing-aid fitting and patient–provider communication Validation status: no formal app validation reported Clinical readiness: clinically deployable/commercial feature under evaluation; used with commercially available ReSound hearing aids and smartphone app Comparator: conventional face-to-face appointment after hearing-aid fitting	Sample size: 30 DHH individuals Target population: DHH adults with mild-to-moderately severe hearing loss and ≥1 year of bilateral hearing-aid experience Age: 22–83 years; mean 67 years Language/sign-language context: English-speaking participants; no sign-language component reported	Usability and acceptability
Fageeh et al., 2024 Front Oral Health [72]	Design: randomized controlled study Country: Saudi Arabia Aim: establish the effectiveness of the use of teledentistry for oral health in DHH and visually impaired individuals	Type: Telesmile mobile application; multimedia Arabic sign-language videos with subtitles Validation status: Telesmile underwent expert evaluation and beta testing Clinical readiness: clinically evaluated; app released on Google Play Store and Apple App Store Comparator: Booklet-based oral hygiene instructions	Sample size: 25 DHH individuals Target population: DHH students Age: 12–18 years; mean 15.6 years Language/sign-language context: Arabic sign language	Oral health and oral-hygiene knowledge assessed using a 14-item questionnaire before and after the 4-week intervention
Kaur et al., 2022 Int J Clin Ped Dent [75]	Design: pilot study Country: India Aim: assess the effectiveness of visual distraction in reducing dental anxiety among DHH and speech children of visual distraction with and without virtual reality glasses	Type: visual distraction without virtual reality glasses; visual distraction with virtual reality glasses Validation status: performance evaluation; no formal validation reported Clinical readiness: clinically evaluated Comparator: no visual distraction	Sample size: 24 DHH children Target population: DHH children who have access to the school dental health program for special needs Age: 6–12 years Language/sign-language context: orally DHH individuals; sign language	Dental and fear anxiety, pulse rate, blood pressure
Liu et al., 2020 Proc ACM Interact Mob Wearable Ubiquitous Technol [87]	Design: pilot feasibility study Country: United States of America Aim: design, evaluate, and demonstrate the feasibility of a low-intrusive, wearable sign language translation system (FinGTrAC)	Type: wearable smart ring and smartwatch Validation status: controlled laboratory validation Clinical readiness: early-stage laboratory prototype; not commercially available or clinically integrated Comparator: prior wearable sign language recognition systems and alternative algorithmic frameworks	Sample size: 10 users Target population: MD Age: MD Language/sign-language context: American sign language	System recognition accuracy, latency/processing time, and system scalability
Lyall et al. 2016 J Laryngol Otol [19]	Design: prospective study Country: United Kingdom Aim: establish the feasibility of communicating using smartphone speech recognition software vs. writing or typing	Type: smartphone speech-to-text application (Dragon Dictation, Apple iPhone 4) Validation status: performance evaluation; no formal validation reported Clinical readiness: clinically evaluated; app released on Apple App Store Comparator: handwriting and computer typing	Sample size: 30 Target population: 11 doctors and 19 medical students communicating with DHH individuals Age: MD Language/sing-language context: MD	Transcription time and accuracy (% words correct); handwriting legibility; correlation between dictation time and accuracy
McLaughlin et al. 2020 BMJ Simul Technol Enhanc Learn [80]	Design: qualitative study Country: United Kingdom Aim: explore medical students’ lived experiences of an immersive virtual reality activity simulating hearing impairment from a patient’s perspective	Type: immersive virtual reality simulation using a headset Validation status: qualitative evaluation Clinical readiness: prototype for medical education Comparator: none	Sample size: 10 Target population: medical students Age: MD Language/sign-language context: not applicable	Students’ lived experiences and perceptions, perceived communication barriers and facilitators, emotional responses, empathy, and reflections
Rakun et al., 2022 IJACSA [77]	Design: prototype and usability study Country: Indonesia Aim: development and evaluation of a mobile text-to-sign system for Indonesian language 3D animation translation system	Type: text-to-sign-language translation system / mobile assistive technology Validation status: performance and usability evaluation Clinical readiness: prototype; not clinically evaluated Comparator: for mouth-movement evaluation: original videos; for combined hand/mouth animation: comparison with hand-gesture-only animation	Sample size: 72 respondents for usability; 6 respondents (3 DHH students and 3 Indonesian sign language teachers for opinion evaluation Target population: DHH students and Indonesian sign language teachers Age: 19–61 years Language/sign-language context: Indonesian sign language	Mean opinion score for mouth-movement quality/understanding and similarity to original videos; usability score; application execution time
Rani et al., 2025 Int J Res Publ Rev [85]	Design: development study Country: India Aim: develop a real-time system to recognize sign language gestures and convert them into text and speech	Type: real-time system developed using machine learning and computer vision techniques Validation status: performance evaluation Clinical readiness: prototype; not clinically evaluated Comparator: no clinical comparator group	Sample size: not applicable Target population: DHH Age: not applicable Language/sign-language context: Indian sign language	Sign-recognition accuracy, precision, recall, real-time latency, and model performance for static versus dynamic gestures
Rhodin et al., 2025 Front Neurol [82]	Design: comparative study Country: Germany Aim: compare different augmented reality glass systems by evaluating their speech-to-text capturing capabilities	Type: augmented reality glasses Validation status: the glasses are commercially available, but they have not undergone formal clinical validation as audiological devices Clinical readiness: for DHH individuals is currently constrained by degraded performance in noisy environments and monosyllabic speech recognition Comparator: four commercial augmented reality systems directly compared with one another	Sample size: 1 Target population: normal-hearing individuals Age: MD Language/sign-language context: not applicable	Speech recognition performance, optical display technology, microphone placement and directionality, system control, connectivity, offline functionality, and app ecosystem integration

## Data Availability

No new data were created or analyzed in this study. Data sharing is not applicable to this article.

## Supplementary Materials

The following supporting information can be downloaded at https://www.mdpi.com/article/10.3390/dj14090589/s1, Supplementary File S1: Classification of Hearing Loss.

## Appendix A

**Table A1:** Advanced search strategy.

Database Filters Date of the Search	Advanced Search Strategy
PubMed/MEDLINE No filters 21 October 2025	((“persons with hearing disabilities”[MeSH Terms] OR (“persons”[All Fields] AND “hearing”[All Fields] AND “disabilities”[All Fields]) OR “persons with hearing disabilities”[All Fields] OR “deaf”[All Fields] OR (“deafness”[MeSH Terms] OR “deafness”[All Fields] OR “deafnesses”[All Fields]) OR “hard-of-hearing”[All Fields] OR “hard-of-hearing”[All Fields] OR “hearing loss”[All Fields] OR “hearing impairment”[All Fields] OR ((“hearing”[MeSH Terms] OR “hearing”[All Fields] OR “hearings”[All Fields]) AND (“disease”[MeSH Terms] OR “disease”[All Fields] OR “disorder”[All Fields] OR “disorders”[All Fields] OR “disorder s”[All Fields] OR “disorders”[All Fields]))) AND (“oral health”[All Fields] OR “dental health”[All Fields] OR (“dentistry”[MeSH Terms] OR “dentistry”[All Fields] OR “dentistry s”[All Fields]) OR “dentist*”[All Fields] OR “dental care”[All Fields] OR “dental disease*”[All Fields] OR “oral disease*”[All Fields] OR “oral prevention”[All Fields] OR “dental treatment”[All Fields] OR “barrier* to care”[All Fields] OR “patient communication”[All Fields] OR (((((“technology”[MeSH Terms] OR “technology”[All Fields] OR “technologies”[All Fields] OR “technology s”[All Fields] OR (“technology”[MeSH Terms] OR “technology”[All Fields] OR “technologies”[All Fields] OR “technology s”[All Fields]) OR “digital intervention*”[All Fields] OR “teledentistry”[All Fields] OR (“telehealth s”[All Fields] OR “telemedicine”[MeSH Terms] OR “telemedicine”[All Fields] OR “telehealth”[All Fields]) OR (“telemedicine”[MeSH Terms] OR “telemedicine”[All Fields] OR “telemedicine s”[All Fields]) OR “digital health”[All Fields] OR (“mhealth s”[All Fields] OR “telemedicine”[MeSH Terms] OR “telemedicine”[All Fields] OR “mhealth”[All Fields]) OR “mobile health”[All Fields] OR “mobile application*”[All Fields] OR “app”[All Fields] OR “virtual reality”[All Fields] OR “augmented reality”[All Fields] OR “tool”[All Fields]) AND “OR”[All Fields]) AND ((“hearing”[MeSH Terms] OR “hearing”[All Fields] OR “hearings”[All Fields]) AND (“assistances”[All Fields] OR “assistant s”[All Fields] OR “assistants”[All Fields] OR “assisted”[All Fields] OR “assisting”[All Fields] OR “assistive”[All Fields] OR “dental assistants”[MeSH Terms] OR (“dental”[All Fields] AND “assistants”[All Fields]) OR “dental assistants”[All Fields] OR “assistant”[All Fields] OR “helping behavior”[MeSH Terms] OR (“helping”[All Fields] AND “behavior”[All Fields]) OR “helping behavior”[All Fields] OR “assist”[All Fields] OR “assistance”[All Fields] OR “assists”[All Fields]) AND (“technology”[MeSH Terms] OR “technology”[All Fields] OR “technologies”[All Fields] OR “technology s”[All Fields]))) AND “OR”[All Fields]) AND ((“visual”[All Fields] OR “visualisation”[All Fields] OR “visualisations”[All Fields] OR “visualise”[All Fields] OR “visualised”[All Fields] OR “visualises”[All Fields] OR “visualising”[All Fields] OR “visualization”[All Fields] OR “visualizations”[All Fields] OR “visualize”[All Fields] OR “visualized”[All Fields] OR “visualizer”[All Fields] OR “visualizers”[All Fields] OR “visualizes”[All Fields] OR “visualizing”[All Fields] OR “visually”[All Fields] OR “visuals”[All Fields]) AND (“teaching”[MeSH Terms] OR “teaching”[All Fields] OR “pedagogies”[All Fields] OR “pedagogy”[All Fields]))))) AND (ffrft[Filter])
Scopus No filters 21 October 2025	(TITLE-ABS-KEY (deaf OR deafness OR “hard of hearing” OR “hard-of-hearing” OR “hearing loss” OR “hearing impairment” OR “hearing disorders”) AND TITLE-ABS-KEY (“oral health” OR “dental health” OR dentistry OR dentist OR “dental care” OR “dental disease” OR “oral disease” OR “oral prevention” OR “dental treatment” OR “barrier to care” OR “patient communication”) OR TITLE-ABS-KEY (technology OR technologies OR “digital intervention” OR teledentistry OR telehealth OR telemedicine OR “digital health” OR mHealth OR “mobile health” OR “mobile application” OR app OR “virtual reality” OR “augmented reality” OR tool OR “hearing assisting technologies” OR “visual pedagogy”))
Web of Science No filters 21 October 2025	TITLE-ABS-KEY = (((deaf OR deafness OR “hard of hearing” OR “hard-of-hearing” OR “hearing loss” OR “hearing impairment” OR “hearing disorders”) AND ((“oral health” OR “dental health” OR dentistry OR dentist* OR “dental care” OR “dental disease*” OR “oral disease*” OR “oral prevention” OR “dental treatment” OR “barrier* to care” OR “patient communication”) OR (technology OR technologies OR “digital intervention*” OR teledentistry OR telehealth OR telemedicine OR “digital health” OR mHealth OR “mobile health” OR “mobile application*” OR app* OR “virtual reality” OR “augmented reality” OR tool” OR “hearing assisting technologies” OR “visual pedagogy”)) (TITLE-ABS-KEY)))
