# Supplementary material for: Oral Health in Deaf and Hard-of-Hearing Individuals: Addressing Widespread Global Health Barriers Through Emerging Digital Technologies—A Comprehensive Review
Source: Dent J (Basel). 2026 Sep 11;14(9):589. doi: 10.3390/dj14090589 (PMC13605037; doi:10.3390/dj14090589)
Supplement: Supplementary file 1 [file dentistry-14-00589-s001.zip › dentistry-4497733-supplementary.pdf]

## Supplementary File S1 – Classification of Hearing Loss

Hearing loss can be classified based on severity, anatomic defect, age of onset, and time of onset in relation to language development, etiology, clinical presentation, frequency loss, and number of ears affected [1,23].

3.1.1. Classification Based on Severity

Hearing loss can be classified based on severity into five categories: mild, moderate, moderately severe, severe, and profound [1]. Individuals with mild-to-moderate hearing loss were typically referred to as "hard-of-hearing", while those with severe or profound hearing loss were more commonly described as "deaf".

3.1.2. Classification Based on Anatomic Defect

In relation to anatomic defects, hearing loss can be divided into three categories: conductive hearing loss (CHL), sensorineural hearing loss (SNHL), and mixed hearing loss [23].

CHL refers to a dysfunction of the external or middle ear, leading to impaired sound transmission to the inner ear, whereas SNHL results from damage to the cochlea, auditory nerve, or central auditory pathways, affecting sound transduction and neural processing [23,24]. Mixed hearing loss is characterized by the coexistence of conductive and sensorineural impairment within the same ear [23].

3.1.3. Classification Based on Age of Onset and Time of Onset in Relation to Language Development

Numerous factors may contribute to the onset of hearing loss, which can occur at different stages throughout the lifespan and can be distinguished into: congenital, prelingual, and postlingual hearing loss, which have significant implications for language development and communication outcomes [1].

During the prenatal period, hearing impairment may arise from genetic factors, including both hereditary and non-hereditary forms, as well as from intrauterine infections such as rubella and cytomegalovirus [1].

In the perinatal period, several conditions have been associated with the development of hearing loss, including birth asphyxia, hyperbilirubinemia, low birth weight, and other perinatal morbidities, as well as complications related to their management [1].

During childhood and adolescence, hearing loss is frequently linked to infectious and inflammatory conditions, particularly chronic ear infections such as chronic suppurative otitis media, as well as the accumulation of fluid in the middle ear, known as chronic nonsuppurative otitis media. Meningitis and other systemic infections may also play a significant role [1].

In adulthood and older age, hearing loss may be associated with chronic diseases, lifestyle-related factors such as smoking, and specific conditions, including otosclerosis, age-related sensorineural hearing loss (presbycusis), and sudden sensorineural hearing loss [1].

Additionally, several factors may contribute to hearing loss across all stages of life [1]. These include cerumen impaction, trauma to the ear or head, prolonged exposure to loud noise, the use of ototoxic medications, occupational exposure to ototoxic chemicals, nutritional deficiencies, viral infections, and other ear-related conditions [1]. Furthermore, some forms of hearing loss may result from delayed-onset or progressive genetic alterations [1].

3.1.4. Classification Based on Etiology

Based on etiology, hearing loss can be distinguished into two major categories: genetic or nongenetic (acquired) [23].

Genetic causes were identified as the causes of about 50%-60% of profound hearing loss in children of developed countries, which occurs in approximately 1 out of every 1,000 newborns [23].

Indeed, around 36% of individuals over 75 years present some degree of hearing im-pairment; in fact, the incidence of hearing loss increases significantly with age [25]. Among these cases, nearly one-third are considered acquired (e.g., ototoxicity, infection, trauma), another third are attributed to genetic inheritance, while the remaining third has an undetermined etiology [25].

#### 3.1.4.1. Genetic Hearing Loss

Hearing loss of genetic etiology can be categorized into simple Mendelian inheritance and complex inheritance [23].

Mendelian forms are further classified as syndromic or nonsyndromic, depending on whether additional anomalies are present [23]. Both syndromic and nonsyndromic types are then subdivided according to their mode of inheritance: autosomal dominant, autosomal recessive, X-linked, or mitochondrial [23,25].

Most genetically determined forms follow an autosomal recessive inheritance pattern (about 70–80%), while autosomal dominant transmission accounts for approximately 15–20% of cases. A smaller proportion, around 2–3%, is associated with X-linked or mitochondrial inheritance [25].

Mitochondrial forms represent a group of conditions characterized by maternal inheritance [23]. Hearing loss associated with mitochondrial inheritance shows a wide range of onset, typically occurring between 5 and 50 years of age [23]. Its severity is variable, although it generally follows a progressive course [23]. In some cases, the etiology of hearing loss results from an interaction between genetic and environmental factors. These disorders typically affect high-energy-demand tissues, including the cochlea [23].

In contrast, complex inheritance involves contributions from both genetic and environmental factors [23].

The number of genes implicated in the development and long-term maintenance of the inner ear has not yet been determined [25]. Current evidence indicates that over 350 genetic conditions have been linked to hearing impairment, with approximately one-third manifesting as syndromic deafness and the majority occurring in non-syndromic forms [25].

I) Syndromic Genetic Hearing Loss

Syndromic SNHL accounts for approximately 30% of congenital hereditary hearing loss and was defined as hearing loss that occurred alongside additional clinical phenotypes, often concerning the eye, skin, kidney, nervous, and musculoskeletal systems [23]. More than 700 syndromes involving hearing loss have been identified, including Pendred syndrome, Alport syndrome, Usher syndrome, Waardenburg syndrome, Jervell and Lange-Nielsen syndrome, Stickler syndrome, Treacher Collins syndrome, branchio-oto-renal syndrome, and CHARGE syndrome [23].

Hearing loss may occasionally be the first symptom occurring in some syndromic forms, with other manifestations presenting later across life [23]. These conditions were referred to as "nonsyndromic hearing loss mimics": for example, type 1 Usher syndrome first presents as congenital profound hearing loss, while the progressive vision loss associated with the syndrome tends to appear later in childhood [23].

Mitochondrial syndromes involving hearing loss have been al identified.

Syndromic characteristics are detailed below:

*Pendred Syndrome*

The most frequent form of syndromic SNHL is Pendred syndrome, occurring in nearly 7.5 per 100,000 individuals, and representing almost 8% of hereditary deafness cases [23]. SLC26A4, encoding for an iodide-chloride symporter known as "pendrin", is the most common mutated gene involved in the genesis of the syndrome [23]. This protein is found in the inner ear, kidneys, and thyroid, and its mutation usually leads to bilateral and profound hearing loss with prelingual onset [23]. On temporal bone imaging, bilateral enlargement of the vestibular aqueducts and cochlear hypoplasia are observed; patients commonly present with euthyroid goiter during their second decade of life [23].

*Usher Syndrome*

Approximately 40 syndromes cause both hearing loss and blindness, with Usher syndrome (USH) being responsible for about half of the cases [23].

The prevalence of Usher syndrome is 1:6,000 to 1:10,000 in the United States and it was subclassified into three variants: USH1, USH2, and USH3 [23].

Patients with USH1 have severe to profound congenital bilateral SNHL and congenital vestibular dysfunction. The ocular manifestation is retinitis pigmentosa, which develops during childhood [23].

Patients with USH2 have moderate to severe congenital SNHL, no vestibular dysfunction, and retinitis pigmentosa that manifests in the third to fourth decade of life [23].

Patients with USH3 have progressive hearing loss, which begins before the third decade, variable vestibular dysfunction, and variable onset of retinitis pigmentosa [23].

*Jervell and Lange-Nielsen Syndrome*

Jervell and Lange-Nielsen Syndrome is an autosomal recessive inherited syndrome, caused by mutations in two potassium channels encoded by the KCNQ1 and KCNE1 genes [23]. The most common manifestations include congenital hearing loss, syncopal attacks, and prolonged QT interval to the electrocardiogram [23].

*Waardenburg Syndrome*

Waardenburg syndrome is an autosomal dominant (types I-III) or recessive (type IV) inherited syndrome, which comprises about 1%-3% of all congenital hearing loss, occurring in 1-2:20,000 people [23].

Typical manifestations include SNHL, dystopia canthorum (lateral displacement of the inner canthi of the eyes) and pigmentary abnormalities of the hair, iris, and skin (e.g., white forelock, heterochromia iridis). Waardenburg syndrome was subclassified into four types: type I, II, III, and IV [23].

Type I presents all of the above-mentioned manifestations and is determined by mutations in the PAX3 gene [23].

Type II is not typically associated with dystopia canthorum [23].

Type III, also called Klein–Waardenburg syndrome, exhibits in addition to type I manifestations, hypoplasia or contracture of the upper extremities and is also caused by mutations in PAX3 [23].

Type IV, also referred to as Waardenburg–Shah syndrome, occurs in conjunction with Hirschsprung disease [23].

*Stickler Syndrome*

Stickler syndrome may be inherited with an autosomal dominant (types I-III) or recessive (types IV-V) pattern, and results from mutations in collagen proteins; however, the underlying pathogenesis is not yet fully understood [23]. Its incidence is 1 in 7500 to 10,000 newborns, with SNHL occurring with aging [23].

Phenotypic manifestations can be rather diverse, as this syndrome can cause sensorineural, conductive or mixed hearing loss [23].

Stickler syndrome was subclassified into five types: types I, II, III, IV and V [23].

Type I, II and III are autosomal dominant and involve the COL2A1, COL11A1, and COL11A2 genes [23].

Types IV and V are autosomal recessive and are determined by mutations in the COL9A1 and COL9A2 genes [23].

*Brachio-Oto-Renal Syndrome*

Brachio-Oto-Renal (BOR) syndrome has an autosomal dominant inheritance pattern and a penetrance of almost 100%, either caused by mutations in the EYA1 gene (nearly 40% of cases), or in the SIX1 and SIX5 genes [23]. BOR syndrome occurs in 1 in 40,000 newborns and is responsible for 2% of profound deafness cases in children [23]. Hearing loss affects about 90% of patients with BOR syndrome and can be sensorineural, conductive, or mixed in nature (20%, 30%, and 50%, respectively); it can be severe in one-third of cases and progressive in one-quarter [23]. The external, middle, and inner ear may all be affected, with several otologic

phenotypes [23]. These include auricular malformations, including microtia and external auditory canal stenosis, preauricular pits or tags, ossicular anomalies, facial nerve dehiscence, absence of the oval window, cochlear hypoplasia, hypoplasia of the lateral semicircular canal, and enlarged vestibular aqueducts [23].

*Treacher Collins Syndrome*

Treacher Collins syndrome is an autosomal dominant condition, while approximately 1% of cases have an autosomal recessive pattern [23]. Its incidence is around 1 in 50,000 individuals, and the clinical presentation is characterized by craniofacial developmental abnormalities, leading to conductive hearing loss secondary to ossicular chain defects in 50% of subjects [23].

*Alport Syndrome*

Alport syndrome has an X-linked, autosomal dominant, or recessive inheritance based on the affected genes [23]. The most commonly involved gene is COL4A5, located on the X chromosome, whereas mutations in two autosomal genes, COL4A3 and COL4A4, are responsible for autosomal dominant and autosomal recessive Alport syndrome [23].

The prevalence is about 1 in 5,000 individuals in the United States and results from mutations in type IV collagen, a primary structural constituent of basement membranes in the kidney, eyes and ear, the organs typically affected [23].

*Mitochondrial Syndrome*

Among the mitochondrial syndromes, several clinical forms have been identified, each characterized by distinct manifestations, including Mitochondrial Encephalopathy, Lactic Acidosis, and Stroke-like episodes (MELAS), Myoclonic epilepsy with ragged red fibers (MERRF), Kearns-Sayre syndrome, Maternally inherited diabetes-deafness (MIDD) syndrome [23].

MELAS syndrome is a mitochondrial encephalopathy associated with lactic acidosis and stroke-like episodes [23].

MERRF syndrome is a myoclonic epilepsy with red ragged fibers [23].

Kearns-Sayre syndrome is characterized by progressive external ophthalmoplegia, pigmentary retinitis and an onset before 20 years of age [23]. Common additional features include deafness, cerebellar ataxia and heart block (available freely online on: https://www.orpha.net/en/disease/detail/480 accessed on 02 February 2026).

MIDD syndrome is a maternally inherited form of diabetes with deafness that occurs in approximately 0.5-2.8% of subjects affected by type 2 diabetes [23].

II) Nonsyndromic Genetic Hearing Loss

The term "nonsyndromic" refers to genetically inherited hearing loss that occurs without any additional phenotypic manifestations [23].

Currently, 63 genetic loci associated with non-syndromic sensorineural hearing loss have been mapped, of which 30 follow an autosomal dominant pattern, 28 are autosomal recessive, and 5 are X-linked [25]. In addition, 19 genes implicated in non-syndromic hearing loss have been successfully cloned, including 7 autosomal recessive genes, 11 autosomal dominant genes, and one X-linked gene [25].

Nonsyndromic SNHL represents nearly 70% of congenital genetic hearing loss, with autosomal recessive being the most common inheritance pattern (about 75%–80%) [23]. Autosomal dominant SNHL accounts for about 20% of cases, while X-linked is associated with less than 2%, and mitochondrial with less than 1% [23].

Autosomal dominant nonsyndromic hearing loss is characterized by postlingual onset (after speech development), a progressive course, and typically a milder degree of hearing loss [23]. The dominant form usually presents distinctive audio profiles: for example, mutations in the KCNQ4 gene, encoding a potassium channel protein, are associated with high-frequency hearing loss; conversely, mutations in the Wolfram syndrome 1 gene result in a typical low-frequency hearing loss, mainly below 2 kilohertz [23].

Autosomal recessive nonsyndromic hearing loss is commonly characterized by prelingual onset (before speech development) and is usually severe-to-profound across all frequencies [23]. Approximately 50% of cases are linked to mutations in GJB2, which encodes connexin 26 [23]. This protein oligomerizes into connexons that form gap junctions between adjacent cells, essential for potassium recycling and cochlear ion homeostasis. Other frequently implicated genes include STRC (16%), SLC26A4 (7%), and TECTA (5%) [23].

X-linked nonsyndromic hearing loss is most commonly associated with mutations in the POU3F4 gene, which encodes a transcription factor [23]. These mutations lead to congenital stapes fixation, cochlear hypoplasia, and enlargement of the lateral internal auditory canal [23]. The resulting hearing loss is typically mixed, and stapes surgery carries a high risk of perilymph gusher [23].

3.1.4.2. Nongenetic (Acquired) Hearing Loss

*Drug-Induced Forms*

Previous evidences shown that over 150 to 600 drugs are associated with ototoxicity adverse effects, which in the short-term of drug therapy should go unnoticed [26]. Early identification of ototoxicity through prospective monitoring allowed timely adjustments in drug therapy and aimed at reducing or preventing permanent sensorineural hearing loss [26]. However, ototoxic effects associated with certain pharmacological treatments may remain unrecognized for long-term periods, as clinicians may not always appreciate the importance of monitoring drug levels [26].

The main drug classes identified as potentially associated with ototoxicity adverse effects included: antibiotics, immunosuppressants, antimalarials, vaccines, antineoplastic agents, nonsteroidal anti-inflammatory drugs, diuretics, and analgesics [26].

In particular, aminoglycoside antibiotics have been strongly associated with increased risk of sensorineural hearing loss because of some variants in the mitochondrial 12S ribosomal RNA (rRNA) gene, MT-RNR1 (A1555G, C1494T, T1095C, 961delT+C(n)), which enhance the resemblance of human 12S rRNA to bacterial rRNA, the molecular target of aminoglycosides [23]. Treatment-induced hearing loss recurred in 2% to 25% of individuals after months following the exposure [23]. The A1555G mutation in MT-RNR1 is associated with a very high risk of hearing loss following aminoglycoside exposure, even after a single dose [23].

*Infectious Causes*

Infectious etiologies play a significant role in explaining the disparities in congenital hearing loss rates between developing and developed countries [23].

Several infectious agents have been associated with the development of hearing loss, both during the prenatal and postnatal periods. The pathogens most commonly implicated in prenatal hearing loss are grouped under the acronym TORCH: Toxoplasmosis, Other (e.g., syphilis), Rubella, Cytomegalovirus (CMV), and Herpesviruses [23].

Congenital CMV infection represents the most frequent non-genetic cause of SNHL worldwide, accounting for 10%-20% of all pediatric cases [23]. According to Goderis et al. [28], hearing loss develops in nearly one-third of symptomatic children, and in about one in ten asymptomatic children with congenital CMV infection. In symptomatic cases, hearing loss is more commonly bilateral, whereas in asymptomatic individuals it tends to be unilateral [28]. Additionally, delayed-onset hearing loss has been reported in children affected by congenital CMV [23].

Prior to the introduction of the rubella vaccination, congenital rubella was the most common viral cause of congenital SNHL [23]. It continues to be in countries without an implemented rubella vaccination program [23,29]. For example, in India, congenital rubella-related hearing loss is estimated to account for 30%–40% of hearing impairment causes, highlighting the important role of vaccination and public health measures in prevention [29].

*Noise-Induced Hearing Loss*

Exposure to loud noise is a known cause of SNHL, especially long-term cumulative noise exposure, whether occupational or recreational [23]. One of the most common occupational diseases is noise-induced hearing loss, representing 16% of impairing adult cases worldwide [30,31]. Recreational noise exposure also represents a significant concern, particularly among adolescents and young adults.
Exposure to loud noise is the most frequent modifiable factor implicated in the development of hearing loss in adults [32]. Based on the intensity and duration of exposure, auditory threshold can be modified temporarily or in a permanent way, with the first sign of noise-induced hearing loss being a threshold shift or notch at 3, 4, or 6 kilohertz [23]. While most cases of noise-induced hearing loss are generally caused by prolonged exposure to moderately intense noise, commonly occurring in occupational and recreational settings, immediate hearing loss can also be caused by acute exposure to very loud noises [23].

*Presbycusis*

Presbycusis, also called "age-related hearing loss," is one of the most common forms of acquired hearing loss [23].
Presbycusis typically occurs within the third decade of life and mostly affects high frequencies, with an overall prevalence of 50.2% in ages 50–59 years and 68.0% in ages 60–69 years [23]. Roth et al. [14] reported that approximately 20% of females and 30% of males in Europe have hearing thresholds exceeding 30 dB at 70 years of age, while by the age of 80, these proportions increase to 45% and 55%, respectively. A considerable group of individuals affected by presbycusis developed late-onset progressive deafness with an underlying genetic predisposition. [25]
In fact, like other age-related conditions, its etiology can be associated with numerous factors, such as genetic predisposition (e.g., sex, ethnicity, genetic variants), cochlear aging, environmental factors (e.g., noise exposure, ototoxic medications), and medical comorbidities (e.g., hypertension, diabetes, stroke, tobacco use) [23].
Moreover, several epidemiological studies have identified an association between hearing loss and dementia in older adults, for which different mechanisms have been suggested [23]. For example, it has been hypothesized that subjects with hearing loss request more cognitive resources for communication, thereby leaving fewer resources available for other mental processes [23]. In this way, hearing loss should result in social isolation, which is a significant risk factor for the development of dementia [23].
Furthermore, according to Thomson et al. [27], the two conditions may share a common underlying etiology (e.g., small vessel disease), with hearing loss potentially representing an early manifestation of the dementia.

*Otosclerosis*

Otosclerosis is a frequent cause of CHL and is characterized by abnormal bone remodeling occurring in the anterior areas of the stapedio-vestibular joint within the otic capsule [23]. This region, known as the fissula ante fenestram [33], undergoes progressive changes that ultimately lead to stapes fixation, resulting in CHL [23].
Bilateral disease is observed in 70%–80% of patients, with a female-to-male ratio of approximately 2:1 [23]. When the disease extends to involve the cochlea, it may cause sensorineural or mixed hearing loss, referred to as cochlear otosclerosis [23]. About 10% of patients with clinical otosclerosis progress to cochlear otosclerosis. The age of manifestation onset of otosclerosis ranges from the late teenage years to the sixth decade, with the mean onset occurring during the third decade of life [23].
Epidemiologic studies distinguish between two forms of otosclerosis: clinical and histological [23].
Clinical otosclerosis is characterized by measurable hearing loss. This form is about 40 times less common in the Caucasian population and is very rare in Asian and Black populations [34,35].
Histological otosclerosis, in which otosclerotic lesions are detected in postmortem temporal bone analyses. This form is found in up to 12.5% of the Caucasian population [36].
The pathogenesis of the disease remains poorly understood; however, both genetic and environmental factors have been implicated [23]. In fact, although otosclerosis was often categorized among acquired forms of

hearing loss, it is now recognized as a multifactorial condition resulting from the interplay between genetic susceptibility and environmental factors [23].

Several genetic pathways, including those involved in bone remodeling and the immune response, have been associated with increased susceptibility to otosclerosis; however, the contribution of each genetic variant is likely small [23]. The inheritance pattern is generally considered autosomal dominant with reduced penetrance, with 40%–50% of cases appearing to be sporadic [23].

Several environmental factors have been associated with the disease, including persistent measles virus infection and estrogen exposure (e.g., through oral contraceptives) [23]. Conversely, exposure to sodium fluoride (e.g., through fluoridated water) has been linked to a protective effect against otosclerosis [23].

#### *3.1.5. Other Classification*

Hearing loss may also be described according to its clinical course in stable, progressive, fluctuating, or sudden, the range of frequencies affected (low, high, or across all frequencies), and the laterality of involvement (unilateral or bilateral).

Together, these classification systems provide a comprehensive framework for understanding the heterogeneity of hearing impairment and its clinical implications.
